# eIF4E1 Regulates *Arabidopsis* Embryo Development and Root Growth by Interacting With RopGEF7

**DOI:** 10.3389/fpls.2022.938476

**Published:** 2022-06-30

**Authors:** Taibo Liu, Qianyu Liu, Zhen Yu, Chunling Wang, Huafu Mai, Guolan Liu, Ruijing Li, Gang Pang, Dingwu Chen, Huili Liu, Jiangyi Yang, Li-Zhen Tao

**Affiliations:** ^1^State Key Laboratory for Conservation and Utilization of Subtropical Agro-bioresources, South China Agricultural University, Guangzhou, China; ^2^Guangdong Provincial Key Laboratory of Protein Function and Regulation in Agricultural Organisms, College of Life Sciences, South China Agricultural University, Guangzhou, China; ^3^Guangdong Laboratory for Lingnan Modern Agriculture, Guangzhou, China; ^4^State Key Laboratory for Conservation and Utilization of Subtropical Agro-bioresources, College of Life Sciences and Technology, Guangxi University, Nanning, China

**Keywords:** auxin, eIF4E1, RopGEF7, embryo development, RAC/ROP, root growth

## Abstract

Eukaryotic translation initiation factor 4E1 (*eIF4E1*) is required for the initiation of protein synthesis. The biological function of *eIF4E1* in plant–potyvirus interactions has been extensively studied. However, the role of *eIF4E1* in *Arabidopsis* development remains unclear. In this study, we show that *eIF4E1* is highly expressed in the embryo and root apical meristem. In addition, *eIF4E1* expression is induced by auxin. *eIF4E1* mutants show embryonic cell division defects and short primary roots, a result of reduced cell divisions. Furthermore, our results show that mutation in *eIF4E1* severely reduces the accumulation of PIN-FORMED (PIN) proteins and decreases auxin-responsive gene expression at the root tip. Yeast two-hybrid assays identified that eIF4E1 interacts with an RAC/ROP GTPase activator, RopGEF7, which has been previously reported to be involved in the maintenance of the root apical meristem. The interaction between eIF4E1 and RopGEF7 is confirmed by protein pull-down and bimolecular fluorescent complementation assays in plant cells. Taken together, our results demonstrated that eIF4E1 is important for auxin-regulated embryo development and root growth. The eIF4E1–RopGEF7 interaction suggests that eIF4E1 may act through ROP signaling to regulate auxin transport, thus regulating auxin-dependent patterning.

## Introduction

RAC/ROP GTPases are multi-functional signaling molecules which regulate a wide range of processes during plant growth and development, including pollen tube growth, leaf epidermal cell morphogenesis, root hair development, embryonic development, seedling growth, stress tolerance, and hormone response (Yang and Fu, 2007; Wu et al., 2011; Craddock et al., 2012; Bloch and Yalovsky, 2013; Li et al., 2021). RAC/ROPs undergo exchange reactions between an active GTP-bound form and an inactive GDP-bound form (Nibau et al., 2013; Akamatsu et al., 2015). The cycling of GTP- and GDP-bound forms is regulated by GTPase-activating proteins (GAPs) and guanine nucleotide exchange factors (GEFs). In short, GAPs catalyze GTP hydrolysis and convert them back to GDP-bound forms. Oppositely, GEFs activate RAC/ROPs by stimulating the exchange of GDP- to GTP-bound forms (Yalovsky, 2015). The plant-specific family of GEFs (i.e., RopGEFs) is utilized predominantly by RAC/ROPs to activate the signaling pathway to regulate plant development (Berken et al., 2005; Chen et al., 2011; Huang et al., 2018).

*Arabidopsis* possesses a distinct small GTPase family of RopGEFs (Gu et al., 2006; Liu et al., 2017). There are 14 *RopGEF* genes in *Arabidopsis*, namely, *RopGEF1* to *RopGEF14*, which are versatile signaling molecules in plant growth and development, as well as responses to various stresses (Wu et al., 2011; Nibau et al., 2013; Liu et al., 2017; Ashraf and Rahman, 2019). The transient expression of *RopGEF1* and *RopGEF12* suggested that these genes mediate polarized pollen tube growth (Gu et al., 2006; Zhang and McCormick, 2007; Chang et al., 2013). In addition, studies of *ropgef1 ropgef4* double mutants and overexpression transgenic lines showed that RopGEF1 and RopGEF4 are involved in ABA-mediated stomatal closure in response to drought stress (Li and Liu, 2012). A recent study reported that RopGEF1 participates in the ABA-mediated inhibition of lateral root growth *via* ABA-mediated protein degradation (Li et al., 2016). Moreover, ABA-induced trafficking and degradation of RopGEF1 are disrupted in a *cpk3/4/6/11* quadruple mutant of calcium-dependent protein kinase (CPK) genes in *Arabidopsis*, suggesting that RopGEF1 cooperates with CPKs and plays crucial roles in root hair polar growth and seedling growth (Li Z. et al., 2018). In our previous study, we have shown that RopGEF1 plays essential roles in the early embryonic and seedling development through regulating polar auxin transport and cell polarity (Liu et al., 2017). Another study reported that phytochrome B (phyB) acts genetically upstream of RopGEF2 and RopGEF4 to activate RopGEF2 regulating stomatal opening (Wang et al., 2017). Our group reported that RopGEF7 participates in regulating auxin-dependent PLETHORA1 (PLT1)- and PLT2-mediated maintenance of root stem cell niches (Chen et al., 2011). In addition, we found that ROP3, which interacts with RopGEF7 (Chen et al., 2011), plays a critical role in maintaining the polarity of auxin efflux proteins (PINs) at the plasma membrane (PM), and whereby regulates embryonic development and postembryonic growth (Huang et al., 2014). To further investigate the role of RopGEF7, we used RopGEF7 as a bait in the screen of *Arabidopsis* seedling cDNA library and identified a potential interaction protein, a translation initiation factor, eIF4E1.

Eukaryotic translation initiation factor 4E (eIF4E) was initially named as the “cap-binding protein” since it interacts specifically with the 5′-terminal cap of mRNA to initiate mRNA translation, a rate-limiting step of protein synthesis (Jackson et al., 2010; Wang and Krishnaswamy, 2012). In *Arabidopsis, eIF4E* comprises a small multiple gene family, including *eIF4E1* (At4g18040, also known as the canonical *eIF4E*), *eIF4E2* (At1g29590, also known as *eIF4E1C*), *eIF4E3* (At1g29550, also known as *eIF4E1B*), *eIF4E1* isoform *eIF(iso)4E* (At5g35620), and *eIF4E* isoforms *nCBP-1* and *nCBP-2* (Patrick et al., 2014; Kropiwnicka et al., 2015; Tajima et al., 2017; Gomez et al., 2019). Published microarray and RNA sequencing data indicate that *eIF4E1* and *eIF(iso)4E* are broadly expressed in various plant tissues at high levels (Zimmermann et al., 2004; Winter et al., 2007), whereas *eIF4E2* and *eIF4E3* are mainly restricted to reproductive tissues and developing embryos at low levels (Zimmermann et al., 2004; Winter et al., 2007; Patrick et al., 2014). Thus, *eIF4E1* and *eIF(iso)4E* show the major activity of this small gene family.

Extensive studies have shown that *eIF4E* genes play an essential role in plant virus infection. Natural mutations in *eIF4E1* and *eIF(iso)4E* enhance the resistance against plant viruses in diverse plant species (Li G. et al., 2018; Liu and Goss, 2018). *ncbp-1/ncbp-2*, a double mutant of the cassava *eIF4E* isoforms *nCBP-1* and *nCBP-2*, displayed delayed and attenuated cassava brown streak disease symptom (Gomez et al., 2019). However, how exactly *eIF4E* mediates plant growth and development has not been well-studied. Downregulation of both *eIF4E1* and *eIF(iso)4E* in antisense tobacco plants caused a semi-dwarf phenotype (Combe et al., 2005). Similarly, silencing of both *eIF4E1* and *eIF4E2* with RNAi strategy in tomato resulted in dwarf plants with fewer seeds and smaller fruits (Mazier et al., 2011). A more pronounced phenotype was observed in the tomato transgenic lines silenced for *eIF4E1, eIF4E2*, and *eIF(iso)4E* (Mazier et al., 2011). The knockout of *eIF(iso)4E* and overexpression of transgenic plants were associated with phosphate-regulated root growth (Martínez-Silva et al., 2012). *Arabidopsis eif4e1* knockout mutants displayed a consistent 7-day bolting delay compared with the wild-type Columbia (Bastet et al., 2018). In this article, we report a specific role for eIF4E1 in embryo development and root growth through interactions with RopGEF7 in the regulation of the auxin pathway.

## Results

### *eIF4E1* Is Expressed in Embryos and Seedlings and Is Induced by Auxin

**Figure 1:**
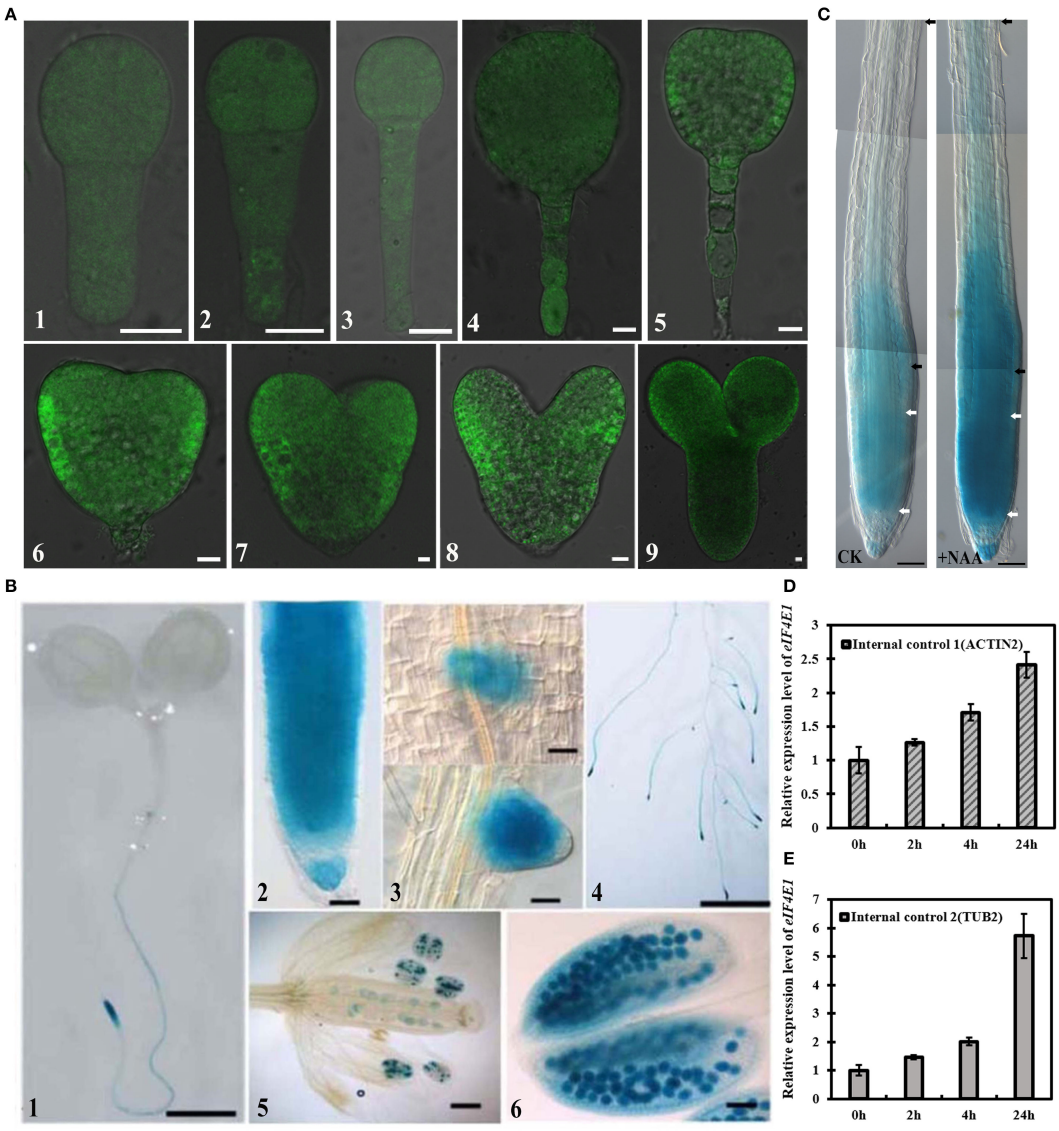
Expression profiles of *eIF4E1* in *Arabidopsis*. **(A)** *eIF4E1*_*pro*_*:YFP-eIF4E1* was expressed in embryos. The confocal images were merged by the DIC and the GFP channels. 8-cell (A1), 16-cell (A2), 32-cell (A3), globular (A4), triangle (A5), early heart (A6, 7), heart (A8), and mature (A9) embryo stages. **(B)** Detection of GUS activity in the primary roots (B1, 2) of 7-day-old *eIF4E1*_*pro*_*:GUS* seedlings, the early stage (up panel) and the late stage (down panel) of lateral root primordia (B3), and lateral roots (B4), as well as in anthers and pollens (B5, 6). **(C)** Detection of GUS activity under exogenous 10 μM NAA treatment. To display the meristem zone and entire elongation zone, **(C)** is generated by three field photographs (using the Photoshop6 software overlap function). The region between two white or two black arrowheads indicates the meristem or elongation zone, respectively. CK, a control without NAA treatment. **(D,E)** Expression level of *eIF4E1* was significantly induced in 7-day-old wild-type seedlings by 10 μM NAA treatment. Transcription levels were normalized to *ACTIN 2* **(D)** or *TUBULIN 2* **(E)**, respectively. Data are presented as mean values of three biological repeats. Scale bars: **(A)** 10 μm; (B1–4) 2 mm; (B5, 6; C) 50 μm.

To investigate the expression patterns of *eIF4E1*, we generated the transgenic plants of *eIF4E1*_*pro*_*:YFP-eIF4E1* with a *YFP* reporter, and *eIF4E1*_*pro*_:*GUS* with a β-glucuronidase (*GUS*) reporter. Analysis of several independent *eIF4E1*_*pro*_*:YFP-eIF4E1* transgenic lines showed that *eIF4E1*_*pro*_*:YFP-eIF4E1* was ubiquitously expressed in both embryo proper and suspensor during embryogenesis. In detail, the YFP signal was detected in 8-cell, 16-cell, 32-cell, globular, triangle, early heart, and heart and mature embryo stages (Figure 1, A1–9), compared to the wild-type embryos which did not show any GFP fluorescence (Supplementary Figure S1). Similarly, *eIF4E1*_*pro*_:*GUS* transgenic lines displayed that GUS activities were expressed in embryogenesis. The GUS activities were detected in early globular, globular, early heart, and heart and mature embryo stages (Supplementary Figure S2). In postembryonic stages, *eIF4E1* was expressed in primary roots (Figure 1, B1, 2; Supplementary Figure S3), lateral root primordia and lateral roots (Figure 1, B3, 4), and anthers (Figure 1, B5, 6), especially with strong expression in pollens (Figure 1, B6). To investigate how *eIF4E1* expression is regulated, we treated the roots of 7-day-old *eIF4E1*_*pro*_:*GUS* seedlings with exogenous auxin NAA. The results indicated that GUS activity was markedly induced by NAA treatment, especially in the meristem region. Compared to untreated control, NAA enlarged the expression area of *eIF4E1* and enhanced GUS activity in the elongation zone of roots (Figure 1C). To confirm such observations, we analyzed the *eIF4E1* relative expression levels by quantitative real-time PCR (qRT-PCR) in 7-day-old wild-type seedling roots. *eIF4E1* expression showed an increase following NAA treatment to up to 24 h; 24-h NAA treatment induced over a two-fold increase in *eIF4E1* relative expression (Figures 1D,E).

### Disruption of *eIF4E1* Results in Developmental Defects During Embryogenesis and Seedling Growth

The expression patterns of *eIF4E1* suggested that *eIF4E1* may be functionally important for embryogenesis. To examine more preciously the role of *eIF4E1, eif4e1-1* carrying a single base replacement mutation which caused a nonsense mutation from Trp (TGG) to a stop codon (TGA), and a T-DNA insertion mutant *eif4e1-2* were used (Supplementary Figure S4A). The relative expression of *eIF4E1* was undetectable in *eif4e1-1* or reduced to <20% in *eif4e1-2*, respectively, compared to those of wild-type Col-0 (Supplementary Figures S4B,C).

**Figure 2:**
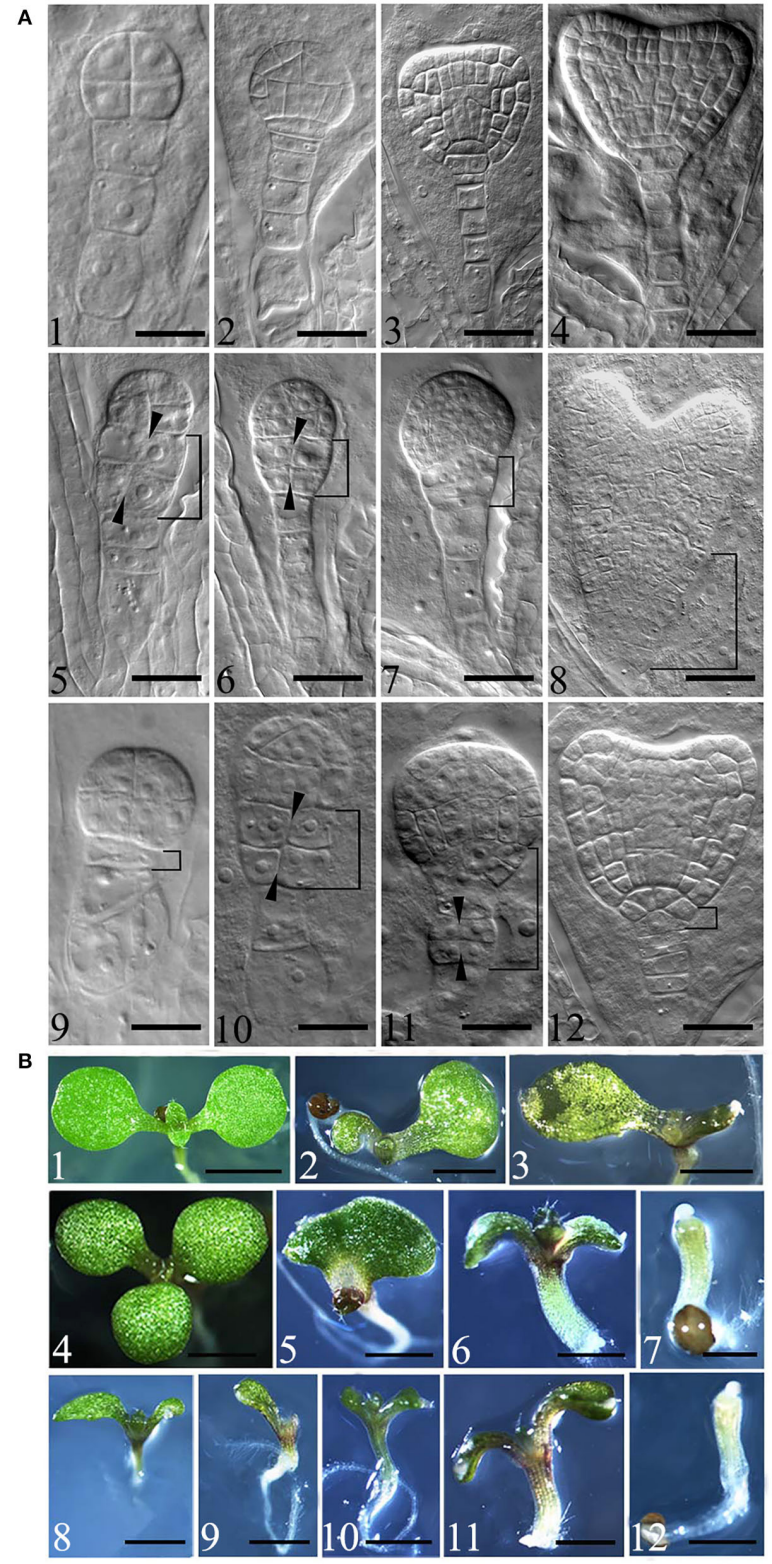
Mutation of *eIF4E1* induces developmental defects in embryo and seedlings. **(A)** Embryo defects in *eif4e1* mutants. Embryos at 8-cell to heart stages from wild type (A1–4), *eif4e1-1* (A5–8), and *eif4e1-2* (A9–12). The black arrowheads show the position of the aberrant cell division plate. The bracketed area indicates cell division defects region. **(B)** Seedling phenotypes of *eif4e1* mutants. Seven-day-old seedlings of wild type (B1), *eif4e1-1* (B2–7), and *eif4e1-2* (B8–12) mutants. Scale bars: **(A)** 50 μm; **(B)** 2 mm.

Embryonic development defects were observed in *eif4e1-1* and *eif4e1-2* from the 8-cell stage. From the 8-cell to early heart stages, *eif4e1-1* and *eif4e1-2* embryos display abnormal divisions in contrast to the wild type (Figure 2, A1–4). *eif4e1* mutants showed abnormal divisions in embryo proper and suspensor, which failed to form a clear boundary between apical and basal regions (Figure 2, A5, 6, 10), as well as an organized root pole (Figure 2, A7, 8, 12). In addition, from the 8-cell to early heart stages, mutant embryos showed aberrant cell divisions in suspensor in *eif4e1-2* (Figure 2, A9, 11); 9.92% of *eif4e1-1* (*n* = 252) and 8.88% of *eif4e1-2* (*n* = 259) embryos displayed embryo developmental defects. The globular stages have the highest defect percentage with 15.15% (*n* = 99) in *eif4e1-1* and 11.02% (*n* = 118) in *eif4e1-2*, respectively, in contrast to wild type, which only had very low percentage of abnormal embryos (1.20%, *n* = 167), as shown in Supplementary Table S2. Taken together, *eIF4E1* is important for embryonic development in *Arabidopsis*, even though only ~10% of embryos showed a defect.

To gain further insights into how *eIF4E1* functions in plant development, we investigated the postembryonic development using 7-day-old seedlings of *eif4e1-1* and *eif4e1-2*. Under normal growth conditions, the wild type has two symmetrical cotyledons and two true leaves (Figure 2, B1). We found that *eif4e1-1* and *eif4e1-2* showed similar phenotypes, for example, developmental defects of cotyledons (Figure 2, B2, 8), deterioration in advance of cotyledons (Figure 2, B3, 9, 10), mutant seedlings with three cotyledons (Figure 2, B4), single cotyledon (Figure 2, B5), extremely short roots (Figure 2, B6, 7, 11, 12), and cotyledon missing (Figure 2, B7, 12). Some of *eif4e1* seedling phenotypes are very similar to auxin mutants (Friml et al., 2003; Blilou et al., 2005), whereas our observations showed that *eif4e1-1* and *eif4e1-2* had 7.74% (*n* = 168) and 6.8% (*n* = 146) developmental defect rates of cotyledon, respectively, as shown in Supplementary Table S3. *eIF4E1* mutants appear to have a similar level of defects (~7%) on seedling cotyledon development in *Arabidopsis*.

### *eIF4E1* Is Necessary for Primary Root Growth

**Figure 3:**
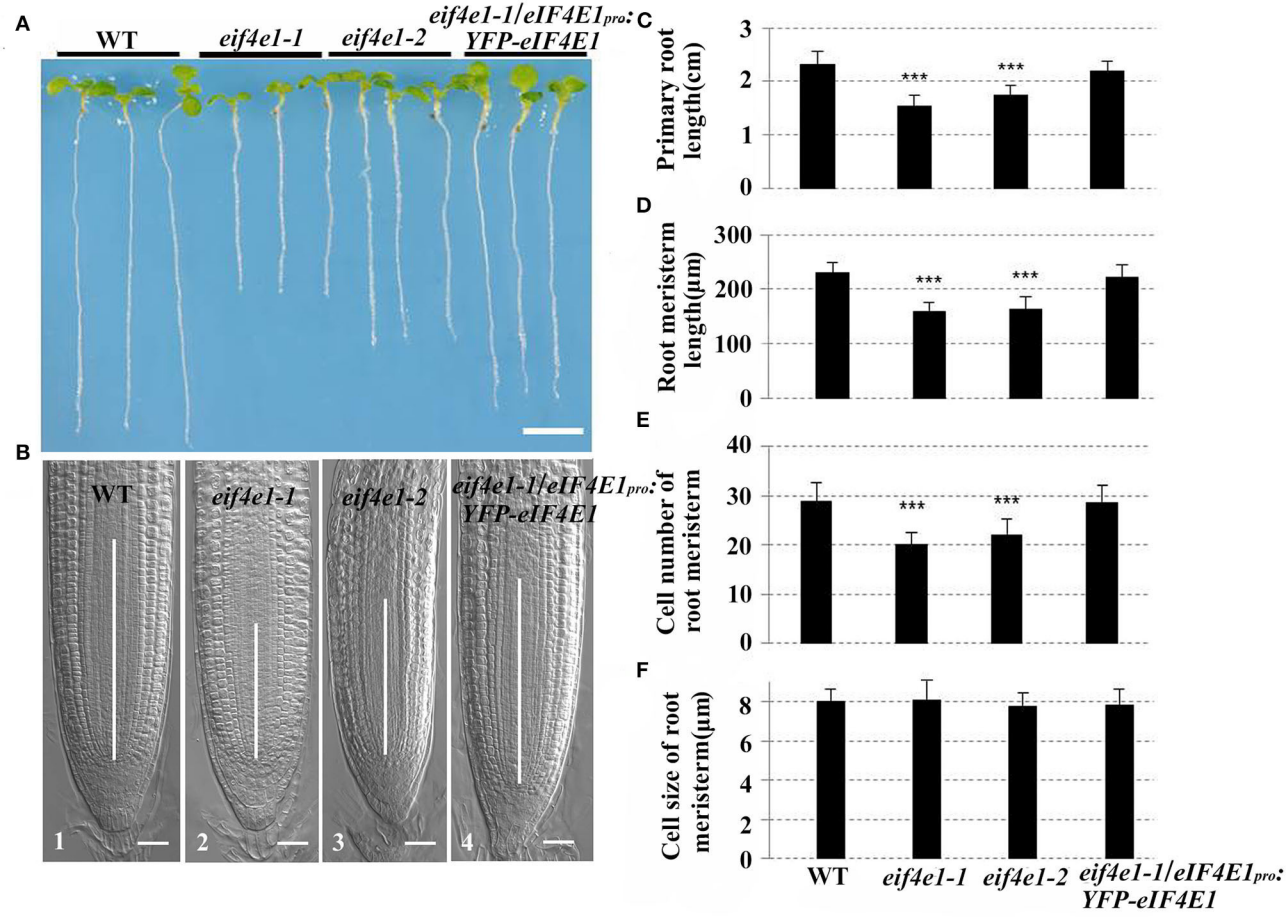
*eIF4E1* mutation affects primary root growth. **(A)** Six-day-old seedlings of wild-type, *eif4e1-1, eif4e1-2*, and complementary line (transgenic plants harboring *eIF4E1*_*pro*_*:YFP-eIF4E1* vector in the *eif4e1-1* background). **(B)** Comparison of RM region of 6-day-old *Arabidopsis* among wild type (B1), *eif4e1-1* (B2), *eif4e1-2* (B3), and *eif4e1-1*/*eIF4E1*_*pro*_*:YFP-eIF4E1* (B4). White lines in the middle of root tips indicate the length of RM. **(C–F)** Root analysis of 6-day-old seedlings. Length of primary root **(C)** and RM **(D)**, cell number of RM **(E)**, and cell size of RM **(F)**. Data are presented as mean values with SD, *n* > 60. The asterisks indicate significant difference by Student's *t*-test (***P < 0.001). Scale bars: **(A)** 4 mm; **(B)** 20 μm.

To investigate the roles of *eIF4E1* in plant root development, we used *eif4e1-1* and *eif4e1-2* to analyze the root growth. The results indicated that the length of primary root of both mutants is remarkably shorter than that of the wild type (Figures 3A,C). To confirm that these phenotypes were caused by the mutation of *eIF4E1*, we generated the complementation transgenic lines by crossing *eif4e1-1* and *eIF4E1*_*pro*_*:YFP-eIF4E1*. The complementary line rescued the shorter primary root phenotype (Figures 3A,C), suggesting that root growth defects were due to loss of *eIF4E1*. To further investigate how *eIF4E1* mutation affects root growth, we measured the length of the root meristem and elongation zones and quantified their cell numbers. The length of root meristem (RM) and elongation zone of the *eif4e1* mutants were significantly shorter than those of the wild type. However, complementary line was able to rescue the short root phenotype of *eif4e1-1* (Figures 3B,D; Supplementary Figure S5). The cell numbers in the meristem and elongation zones of mutants were less than those of the wild type but recovered in the complementary transgenic line (Figure 3E; Supplementary Figure S5). We could not, however, see any difference in the cell size of the root apical meristem between *eif4e1* mutants and wild type (Figure 3F), suggesting that *eIF4E1* may regulate root growth by affecting cell division of root cells.

### eIF4E1 Regulates Auxin-Responsive Report Gene Expression and the Accumulation of PIN3 and PIN7 Proteins

**Figure 4:**
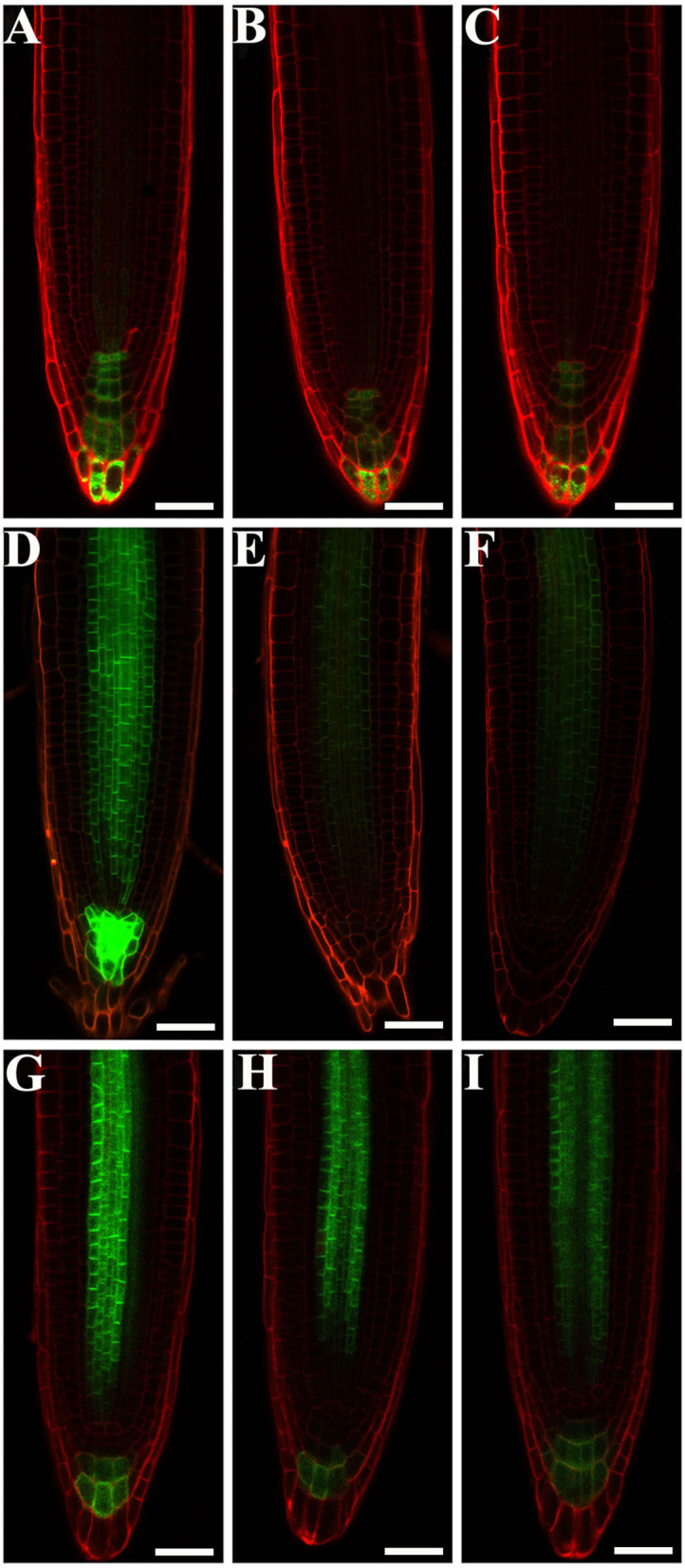
eIF4E1 regulates the expression of auxin-responsive report gene and the accumulation of PIN3 and PIN7 proteins. **(A–C)** *DR5*_*rev*_*:GFP* is expressed in roots of 4-day-old wild type **(A)**, *eif4e1-1* **(B,C)**. “L1” and “L2” designate two lines of *eif4e1-1* crossed by *DR5*_*rev*_*:GFP*. **(D–F)** *PIN3*_*pro*_*:PIN3-GFP* is expressed in roots of 4-day-old wild type **(D)**, *eif4e1-1* **(E,F)**. “L1” and “L2” designate two lines of *eif4e1-1* crossed by *PIN3*_*pro*_*:PIN3-GFP*. **(G-I)** *PIN7*_*pro*_*:PIN7-GFP* is expressed in roots of 4-day-old wild type **(G)**, *eif4e1-1* **(H,I)**. “L1” and “L2” designate two lines of *eif4e1-1* crossed by *PIN7*_*pro*_*:PIN7-GFP*. Scale bars: 20 μm.

The developmental phenotypes of the embryo and seedling of *eif4e1* mutants (Figures 2A,B), and the expression levels of *eIF4E1* were induced by exogenous NAA application (Figures 1D,E), suggesting that *eIF4E1* may participate in the regulation and/or expression of auxin-mediated processes. To test our speculation, we examined whether auxin reporter *DR5*_*rev*_*:GFP* and auxin efflux carrier reporters were altered in *eif4e1* mutants. First, we crossed *DR5*_*rev*_*:GFP* transgenic plants with *eif4e1-1* mutants. We then examined DR5 activity during root growth in *eif4e1-1* andcompared with that of the wild type. Two independent lines, L1 (Figure 4B) and L2 (Figure 4C), showed markedly reduction in GFP fluorescent signals in contrast to the wild type (Figure 4A), suggesting that *eIF4E1* participates in auxin-regulated root growth.

Auxin efflux carrier PIN proteins are major players to generate auxin gradients in root tips (Adamowski and Friml, 2015; Sauer and Kleine-Vehn, 2019). To investigate the *PIN* expression in *eif4e1* mutants, we crossed the *PIN3*_*pro*_*:PIN3-GFP* and *PIN7*_*pro*_*:PIN7-GFP* into *eif4e1-1*, respectively. We next analyzed the expression of *PIN3*_*pro*_*:PIN3-GFP* and *PIN7*_*pro*_*:PIN7-GFP* in the roots of the *eif4e1-1* mutant. In wild-type roots, PIN3-GFP is localized in root stele cells and the root cap, while the PIN3-GFP signal was strongly reduced in *eif4e1-1* L1 (Figure 4E) and L2 (Figure 4F) roots. Compared with the wild type, PIN3-GFP was even absent in the root cap (Figure 4D). We also found that PIN7-GFP was reduced in the roots of two independent *eif4e1-1* lines (Figures 4H,I) compared to the wild type (Figure 4G), which further supports the importance of PIN3 and PIN7 in forming auxin gradients. We also examined the transcription level of *PIN3* and *PIN7* in *eif4e1* mutants, and a slight elevation in the mutant background was observed compared to the wild type (Supplementary Figure S6). However, the location and accumulation of PIN1-GFP (Supplementary Figures S7B,C), PIN2-GFP (Supplementary Figures S7E,F), and AUX1-YFP (Supplementary Figures S7H,I) were not affected in *eif4e1-1* in contrast to the wild type (Supplementary Figures S7A,D,G). Taken together, the loss-of-function mutation of *eIF4E1* obviously reduced the expression of *DR5*_*rev*_*:GFP* and the abundance of PIN3 and PIN7 proteins but has no effect on the localization and accumulation of PIN1, PIN2, and AUX1.

### Treatment of CHX Can Mimic the Phenotypes of *eif4e1*

**Figure 5:**
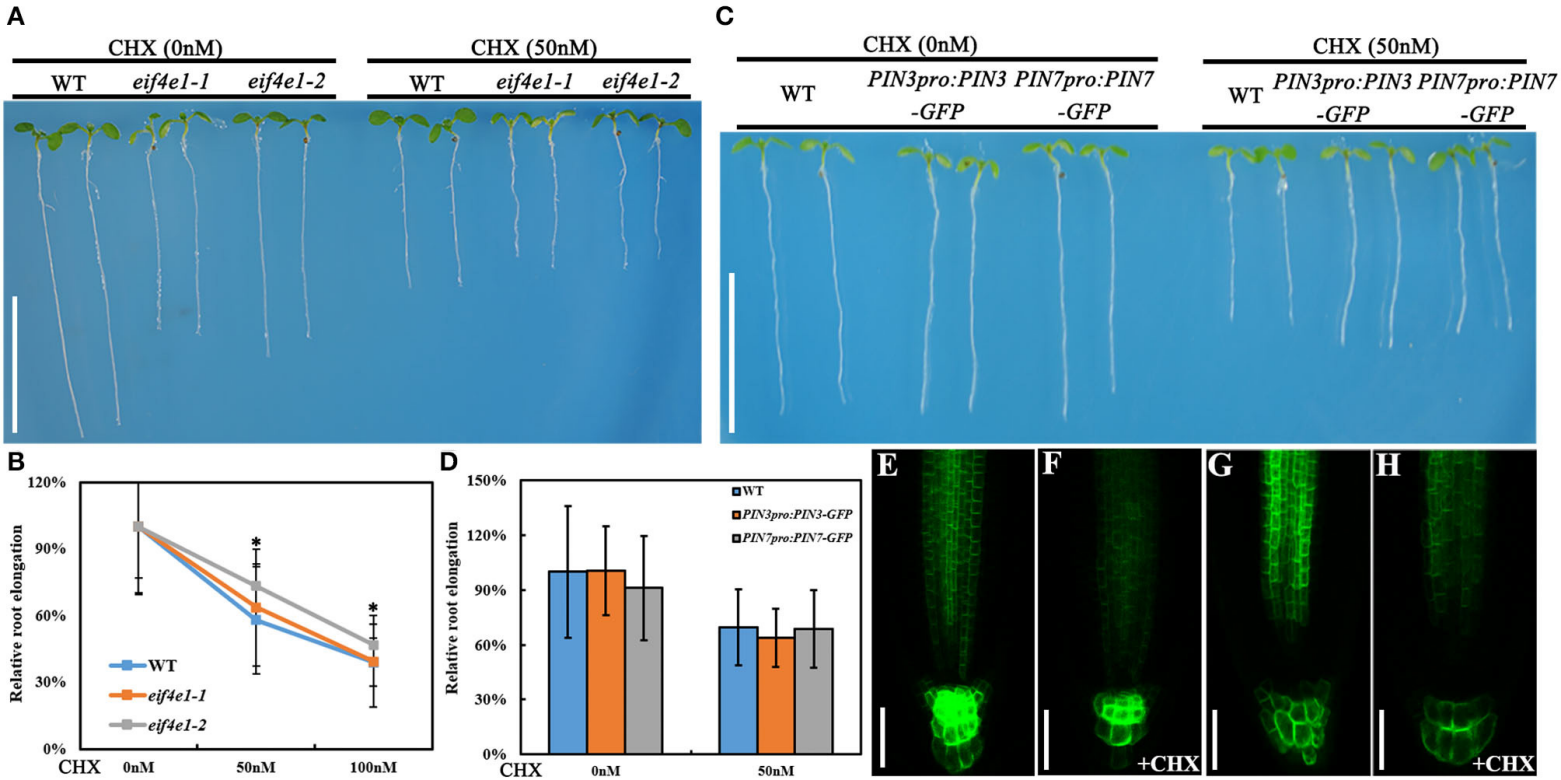
CHX treatment can mimic the *eif4e1* phenotypes. **(A)** CHX treatment inhibits the root elongation. Four-day-old seedlings of wild type, *eif4e1-1*, and *eif4e1-2* were transferred onto half-strength MS medium with or without 50 nM CHX for another 3-day growth. **(B)** Relative root elongation of wild type, *eif4e1-1*, and *eif4e1-2* after treated with 50 or 100 nM CHX. **(C)** Four-day-old seedlings of wild type, *PIN3*_*pro*_*:PIN3-GFP*, and *PIN7*_*pro*_*:PIN7-GFP* were transferred onto half-strength MS medium with or without 50 nM CHX for another 3-day growth. **(D)** Relative root elongation of **(C)**. **(E–H)** Confocal images showing the abundance of PIN3 and PIN7 proteins at the root cells of 4-day-old seedlings. Four-day-old seedlings of *PIN3*_*pro*_*:PIN3-GFP* and *PIN7*_*pro*_*:PIN7-GFP* were treated with **(F,H)** or without **(E,G)** CHX for another 3-day growth. The asterisks indicate significant difference by Student's *t* test (*P < 0.05). Scale bars: **(A,C)** 1 cm; **(E–H)** 50 μm.

Considering that eIF4E1 is a eukaryotic translation initiation factor, we examined whether the treatment of translation inhibitor cycloheximide (CHX) can mimic the phenotypes of *eif4e1* mutants. *eif4e1* mutants and the wild type exhibit significant inhibition in root elongation in the presence of 50 nM CHX, compared to those without CHX treatment (Figures 5A,B), and *eif4e1* mutants were less sensitive to CHX than the wild type (Figures 5A,B). In view of the decline of PIN3 and PIN7 protein abundance in *eif4e1* mutants, we investigated whether the CHX treatment affects PIN accumulation. Seedlings harboring *PIN3*_*pro*_*:PIN3-GFP* or *PIN7*_*pro*_*:PIN7-GFP* display inhibited root growth similar to the wild type (Figures 5C,D). Moreover, the abundance of PIN3 and PIN7 proteins is reduced in the presence of CHX (Figures 5F,H), compared to that of CHX treatment (Figures 5E,G). Meanwhile, we examined the abundance of PIN1, PIN2, and AUX1 proteins after CHX treatment, and we also could see slight alterations on protein abundance compared to those without CHX treatment (Supplementary Figure S8). Taken together, our observations showed that the treatment of CHX can mimic the phenotypes of *eif4e1*, suggesting that root growth defects of *eif4e1* mutants are attributed to altered polar auxin transport.

### eIF4E1 Interacts With RopGEF7

**Figure 6:**
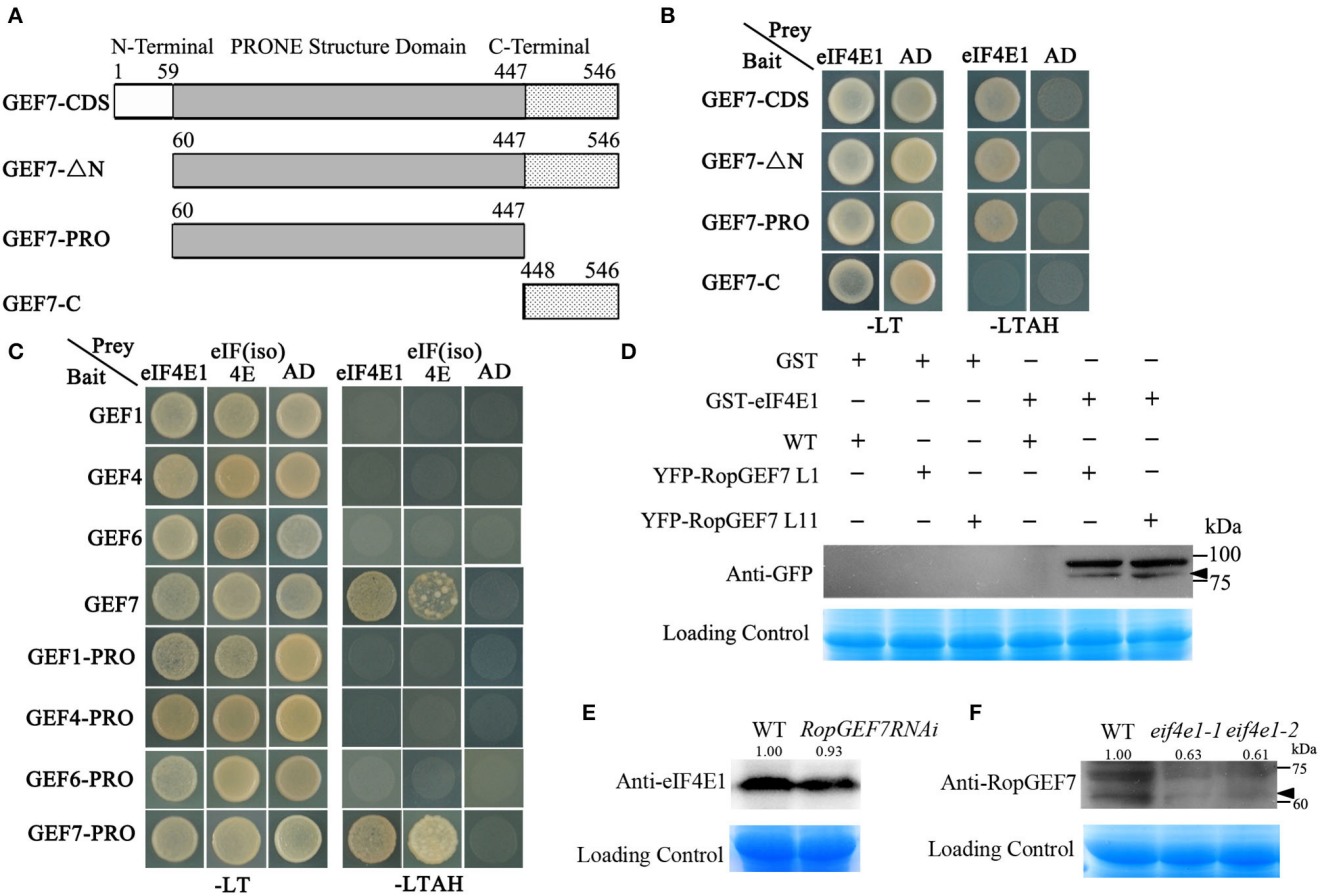
eIF4E1 interacts with RopGEF7. **(A)** Schematic representation of the different truncated versions of RopGEF7. **(B)** Yeast two-hybrid assay showed that eIF4E1 interacted with RopGEF7-CDS, -ΔN, and -PRONE, respectively. SD/-LT indicates SD/-Leu-Trp; SD/-LTAH indicates SD/-Leu-Trp-Ade-His. **(C)** Yeast two-hybrid assay showed that eIF4E1 and eIF(iso)4E interacted with RopGEF7-CDS and -PRONE, respectively. **(D)** Pull-down assays indicate the interactions between eIF4E1 and RopGEF7. *35S*_*pro*_*:YFP-RopGEF7* indicates *35S*_*pro*_*:YFP-RopGEF7* transgenic plants. L1 and L11 indicate two individual *35S*_*pro*_*:YFP-RopGEF7* transgenic lines. GFP antibody was used for detection. **(E)** Abundance of eIF4E1 protein in *RopGEF7 RNAi* transgenic line was detected by Western blotting. Anti-eIF4E1 was designated as eIF4E1 antibody. **(F)** Abundance of RopGEF7 protein in *eif4e1-1* and *eif4e1-2* was detected by Western blotting. Anti-RopGEF7 was designated as RopGEF7 antibody. The protein amount was quantified by its loading control for each sample, and the protein value of the wild type was set as one. The values are shown up the bands in **(E,F)**. The black arrowheads indicate the aim protein bands in **(D,F)**.

**Figure 7:**
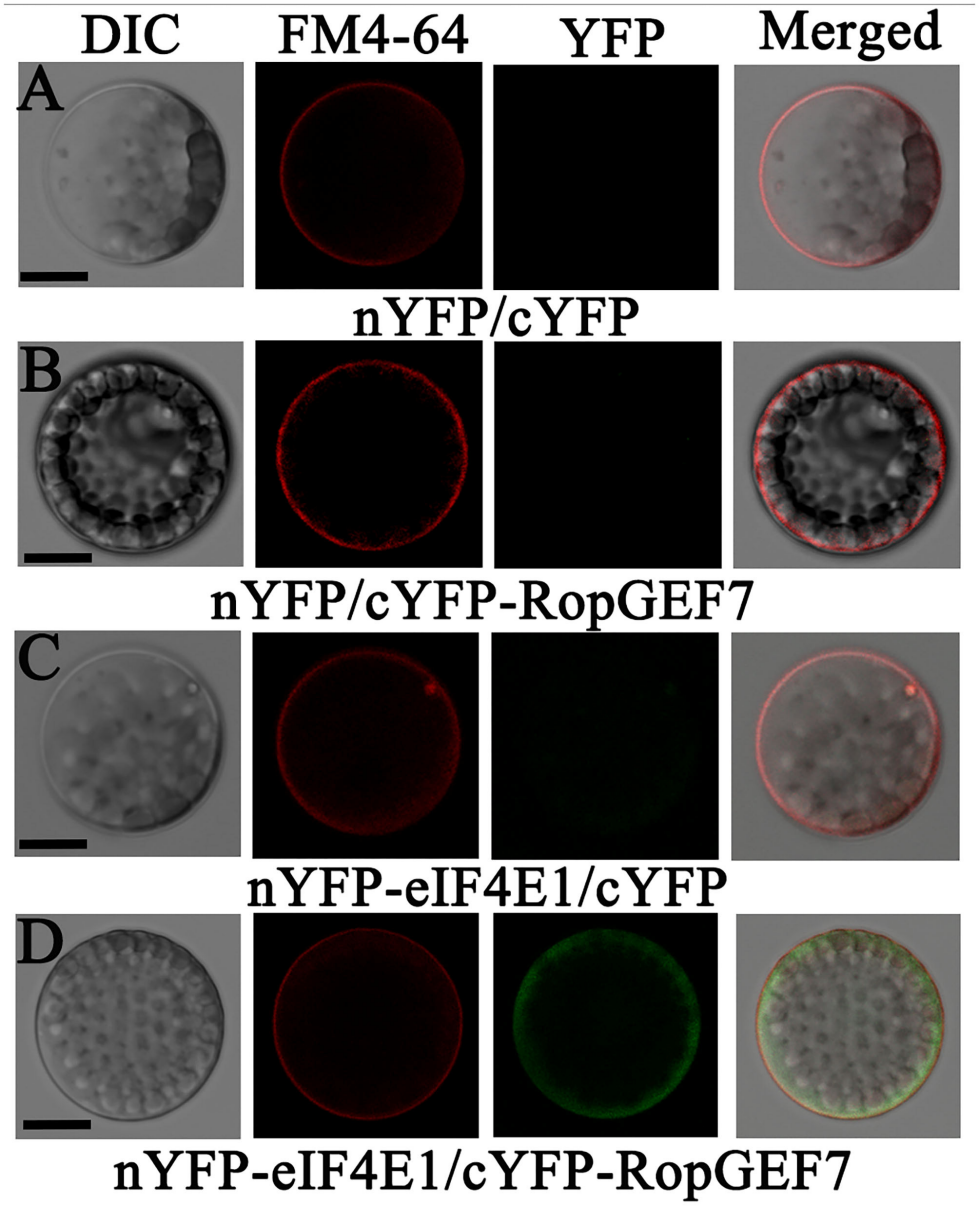
eIF4E1 interacts with RopGEF7 in *Arabidopsis* protoplasts. **(A–D)** *Arabidopsis* protoplasts were co-transfected with **(A)** N-terminal *YFP* and C-terminal *YFP*, **(B)** *nYFP* and *cYFP-RopGEF7*, **(C)** *nYFP-eIF4E1* and *cYFP*, and **(D)** *nYFP-eIF4E1* and *cYFP-RopGEF7*. The FM4-64 dye labels the plasma membrane. Scale bars: 20 μm.

**Figure 8:**
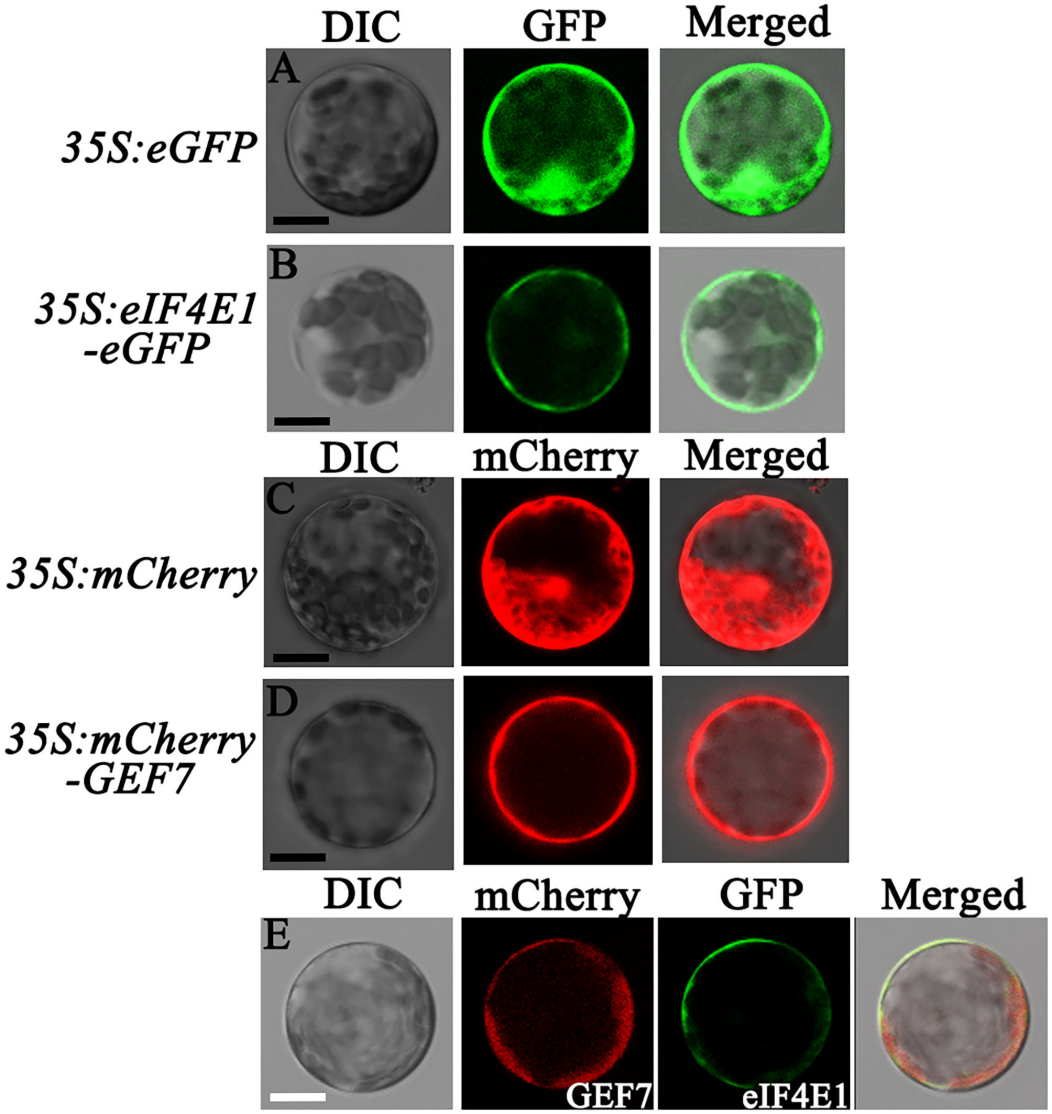
Subcellular localization of eIF4E1 and RopGEF7 in *Arabidopsis* protoplasts. (A-D) Subcellular localization of GFP from a cell harboring a *35S:eGFP* empty vector **(A)**, GFP-tagged eIF4E1 in a *35S:eIF4E1-eGFP* expressed cell **(B)**, mCherry in a cell expressed *35S:mCherry* empty vector **(C)**, and mCherry-labeled RopGEF7 in a *35S:mCherry-RopGEF7* expressed cell **(D)** in *Arabidopsis* protoplasts, respectively. **(E)** Co-localization of eIF4E1-eGFP and mCherry-RopGEF7 in *Arabidopsis* protoplasts. Scale bars: 20 μm.

*AtRopGEF7* is an activator of ROP GTPases, molecular switches function in multiple signaling pathways (Chen et al., 2011; Yalovsky, 2015). Previously, our group reported that *AtRopGEF7* regulates the maintenance of the root apical meristem *via* affecting PIN-dependent polar auxin transport (Chen et al., 2011; Huang et al., 2014). Interestingly, we found a potential interacting eIF4E1, when we used RopGEF7 as bait in the screen of *Arabidopsis* seedling cDNA library. The cap-binding translation initiation factor, eIF4E1, could be involved in a potential RopGEF7 regulatory mechanism. To further confirm the interaction between eIF4E1 and RopGEF7, we performed the Y2H direct interaction assay. eIF4E1 strongly interacted with full-length protein of RopGEF7 (RopGEF7-CDS) and truncated proteins containing the PRONE domain (RopGEF7-ΔN and RopGEF7-PRONE), respectively, whereas eIF4E1 could not interact with the C terminus of RopGEF7 (RopGEF7-C) (Figures 6A,B), suggesting that the interaction requires a functional PRONE structure domain. Furthermore, we found eIF(iso)4E, a homolog of eIF4E1, also interacted with the PRONE domain of RopGEF7 but only weakly interacted with the full length of RopGEF7 in yeast cells (Figure 6C), suggesting this interaction may differ between these two forms of the cap-binding protein. Additionally, considering the conserved PRONE domain of RopGEFs, we tested whether the three homologs RopGEFs (RopGEF1, RopGEF4, and RopGEF6) closely related to RopGEF7 interact with eIF4E1 in yeast cells. However, eIF4E1 does not interact with them (Figure 6C), suggesting that eIF4E1 may predominantly interact with RopGEF7. The interaction between eIF4E1 and RopGEF7 was further confirmed by pull-down assays. In these experiments, the recombinant GST-tagged eIF4E1 protein and the YFP-RopGEF7 total plant protein were used as bait and prey, respectively. GFP antibody was used to detect the interaction between eIF4E1- and YFP-tagged RopGEF7 proteins. The results further support the interaction between eIF4E1 and RopGEF7 (Figure 6D). Moreover, to further investigate the relationship between RopGEF7 and eIF4E1, by using RopGEF7 antibody, the immunoblotting analysis showed that the abundance of RopGEF7 protein in *eif4e1-1* and *eif4e1-2* was markedly reduced (Figure 6F). On the contrary, the level of eIF4E1 protein in *RopGEF7 RNAi* transgenic lines was not affected (Figure 6E). To further verify the interactions between eIF4E1 and RopGEF7 in plant cells, we performed bimolecular fluorescence complementation (BiFC) assays in *Arabidopsis* protoplasts. The BiFC constructs of *eIF4E1* in combination with *RopGEF7* were co-transformed into *Arabidopsis* protoplasts, and the BiFC-generated yellow fluorescent protein (YFP) signal was observed in the cortical cytoplasm (Figure 7D), whereas the controls showed only the background signal (Figures 7A–C). We next observed the subcellular localization of RopGEF7 and eIF4E1. The results indicated that GFP-tagged eIF4E1 fusion protein was localized at the cytoplasm (Figure 8B), and mCherry-labeled RopGEF7 was localized at the cytoplasm and PM (Figure 8D), compared to their GFP or mCherry control vectors which are widely expressed at nucleus, cytoplasm, and PM (Figures 8A,C). Moreover, subcellular localization of YFP-eIF4E1 in roots of *eIF4E1*_*pro*_:*YFP-eIF4E1* transgenic plants displayed that YFP-eIF4E1 is predominantly localized at the cytoplasm (Supplementary Figure S3). Meanwhile, co-transformation of *35S:eIF4E1-eGFP* and *35S:mCherry-RopGEF7* in *Arabidopsis* protoplasts showed obviously merged signals in the cytoplasm (Figure 8E), indicating that eIF4E1 and RopGEF7 are colocalized at the cytoplasm. Taken together, these results show that eIF4E1 interacts with RopGEF7 and may act on the upstream of RopGEF7 to regulate RopGEF7 protein translation.

## Discussion

Previous investigations revealed the important role of eIF4E1 in plant–potyvirus interactions (Wang, 2015). Little is known about its function in plant growth and development. In this work, we show that mutations in *eIF4E1* resulted in embryo defects and reduced root growth (Figures 2, 3). The developmental abnormalities of *eif4e1* might be attributed to altered polar auxin transport based on the auxin marker analysis (Figure 4). Furthermore, we discovered that eIF4E1 interacts directly with RopGEF7, an activator of ROP GTPases to regulate the abundance of RopGEF7 protein (Figure 6). Our results provide compelling evidence that eIF4E1 regulates auxin-dependent development and also establishes a link between protein translation and ROP signaling.

We found that *eIF4E1* is broadly expressed in embryos from the early embryo stage to the mature embryo stage (Figure 1, A1–9), and the *eif4e1* mutant embryos showed cell division defects at embryo proper, basal region, and suspensor from the 8-cell stage to the mature embryo stage (Figure 2A; Supplementary Table S2). *RopGEF7* is also expressed in embryos, but its expression did not fully overlap with *eIF4E1*; it is rather restricted to the precursor cells of the quiescent center (QC) during the early heart stage to the mature stage of embryogenesis (Chen et al., 2011). The knockdown of *RopGEF7* by RNA interference technique caused the defects in the root pole and suspensor (Chen et al., 2011). Interestingly, the expression pattern for *RopGEF1*, a close homolog of *RopGEF7*, is comparable to that of *eIF4E1* in the embryo (Liu et al., 2017). Moreover, the phenotypes of the embryo of *ropgef1* resemble to those of *eif4e1* mutants (Liu et al., 2017).

To further understand the role of *eIF4E1* in plant early development, we characterized a point mutation mutant *eif4e1-1* (*cum1*) and a T-DNA insertion mutant *eif4e1-2* (Supplementary Figure S4). Both mutant lines displayed embryo phenotypes and seedling cotyledon phenotypes (Figure 2), which partially originated from defective embryo development. These data indicates that *eIF4E1* is important for embryo development. Further study to examine whether *eIF(iso)4E*, an isoform of *eIF4E1*, contributes to embryo development would provide more information. Several genetic studies failed to generate *eif4e1 eif(iso)4e* double mutants but only obtained plants homozygous for one locus and heterozygous for the other locus, due to embryo or gametophyte lethality (Callot and Gallois, 2014; Patrick et al., 2014).

The strong expression of *eIF4E1* in the root apical meristem (Figure 1, B1; Supplementary Figure S3) supports its importance in root development. The expression pattern in the root apical meristem shown by *eIF4E1*_*pro*_*:GUS* reporter (Figure 1) is consistent with previous investigation by mRNA *in situ* hybridization (Rodriguez et al., 1998). *eIF4E1* expression is induced by auxin (Figures 1C–E), suggesting that it might function in the auxin-regulated pathway. *eif4e1* mutants developed shorter roots with small size of root apical meristems and elongation zones than those of the wild type (Figure 3; Supplementary Figure S5). Auxin maxima were reduced in the *eif4e1* root tips (Figures 4A–C). Auxin efflux carrier PIN proteins are major contributors to establish the auxin maxima in the root tips. We also observed the abundance of PIN3 and PIN7 to be reduced in *eif4e1* mutants (Figures 4D–I). However, the transcript level of *PIN3* and *PIN7* did not decrease in *eif4e1* mutants (Supplementary Figure S6). We cannot exclude the possibility that eIF4E1 directly affects PIN protein translation. CHX treatment resulted in reduced root growth (Figures 5C,D) and a decreased abundance of PIN3 and PIN7 proteins (Figures 5E–H). Further study is required to decipher the precise mechanism how eIF4E1 affects the PIN accumulation and auxin-dependent development.

Our previous work showed that the knockdown of *RopGEF7* by RNA interference technique caused defects in root growth and failure to maintain root stem cell niche (Chen et al., 2011). RopGEF7 could activate ROP GTPases, including ROP3, which is required for the localization of several PIN proteins in the plasma membrane of root cells (Chen et al., 2011; Huang et al., 2014). *RopGEF7RNAi* plants and *ROP3-*dominant negative plants showed reduced auxin maxima reflected by the *DR5*_*rev*_*:GFP* auxin reporter in the root tips (Chen et al., 2011; Huang et al., 2014). An ROP effector ICR1 is required for the recruitment of PIN proteins to the PM, and loss of function in *ICR1* resulted in the reduced auxin maxima in the root tips and caused the collapse of the root apical meristem (Hazak et al., 2010). Interestingly, ICR1 induced by auxin is essential for auxin transport. When auxin reaches certain levels, ICR1 is degraded to result in auxin accumulation in the embryo hypophysis and the root tips (Hazak et al., 2014). In *eif4e1* mutants, the perturbed auxin maxima in the root tips (Figures 4A–C) likely led to the retarded root growth. At present, it remains unclear how eIF4E1 acts through ROP signaling to affect auxin transport; additional work will be needed to elucidate how eIF4E1 regulates RopGEF7 to activate ROP in the plant cells. A recent study has shown that RALF1 peptide hormone promotes FERONIA receptor kinase-mediated phosphorylation of eIF4E1, which modulates mRNA translation and root hair polar growth (Zhu et al., 2020). FERONIA is also known to activate ROP signaling by interacting with several RopGEF1/4/7/10 to regulate auxin-dependent root hair development (Duan et al., 2010). Our recent study showed that FERONIA targeted PIN2 polarity to regulate lateral root growth and root gravitropic response (Dong et al., 2019). It is likely that FERONIA, eIF4E1, and RopGEF7 may act on the same pathway to regulate PIN-dependent auxin transport, which is essential for embryo and root development. Taken together, we provide evidence that eIF4E1 plays a critical role in embryo development and root growth in *Arabidopsis*. Mutations in *eIF4E1* caused aberrant cell divisions in embryos and reduced cell numbers of the root apical meristem that might be attributed in part to the altered polar auxin transport. Moreover, eIF4E1 physically interacts with RopGEF7 (Figures 6B–D) to regulate the accumulation of RopGEF7 protein (Figure 6F) for targetingauxin-mediated embryo and root development in *Arabidopsis*.

## Materials and Methods

### Plant Materials and Growth Conditions

*Arabidopsis eif4e1-1* (CS6552) and *eif4e1-2* (SALK_145583C) mutants were provided by ABRC (Ohio State University). *eif4e1-1* carrying a single base replacement mutation caused a nonsense mutation from Trp (TGG) to a stop codon (TGA), and *eif4e1-2* is a T-DNA insertion mutant. All *Arabidopsis* mutants and transgenic lines were derived from ecotype Col-0. Marker lines were used as previously described: *DR5*_*rev*_*:GFP* (Benková et al., 2003), *PIN2*_*pro*_*:PIN2-GFP* (Blilou et al., 2005), *PIN3*_*pro*_*:PIN3-GFP* (Blilou et al., 2005), *PIN7*_*pro*_*:PIN7-GFP* (Blilou et al., 2005), and *AUX1*_*pro*_*:AUX1-YFP* (Swarup et al., 2004). Hybridization lines were obtained by crossing *eif4e1-1* with the aforementioned marker lines, respectively. Seeds were surface-sterilized and germinated on half-strength Murashige and Skoog (MS) medium supplemented with 1.0% sucrose and were treated at 4°C for 2–3 days in the dark for synchronization and transferred to growth chambers at 22°C under long-day conditions (16-h light/8-h dark cycle). Then 7-day-old seedlings were transferred to soil and grown under the same conditions (Huang et al., 2014; Liu et al., 2017).

### Plasmid Construction and Plant Transformation

To construct a vector for the *eIF4E1*_*pro*_:*GUS* transgenic plants, a fragment of the 3,926-bp *eIF4E1* promoter sequence was cloned into pBI101.1 at *Hin*dIII and *Bam*HI sites. To generate *35S*_*pro*_*:YFP-RopGEF7* transgenic plants, a constitutive promoter from *cauliflower mosaic virus* (*CaMV*) was used, and two independently transgenic lines, *35S*_*pro*_*:YFP-RopGEF7 L1* and *L11* with high levels of YFP-RopGEF7 expression, were obtained. *eIF4E1*_*pro*_:*YFP-eIF4E1* vector contains a YFP coding sequence (CDS) fused in-frame with an *eIF4E1* CDS under the control of the same 3,926-bp *eIF4E1* promoter region.

The aforementioned constructs were introduced into *Agrobacterium tumefaciens* strain GV3101 and subsequently used to transform *Arabidopsis* (Col-0) by using the floral dip method (Clough and Bent, 1998). The transgenic plants were screened on half-strength MS agar medium containing 25 μg/mL kanamycin or 15 μg/mL hygromycin. The primers used for generating the constructs are listed in Supplementary Table S1.

### Quantitative RT-PCR Analysis

For qRT-PCR analysis, total RNA was extracted from 7-day-old seedlings using the RNeasy plant mini kit (Qiagen), and the first-strand cDNA was synthesized using the Takara PrimeScript First-strand cDNA synthesis kit (TAKARA). Next, PCR was performed on an Illumine Eco (Illumina) System with an SYBR probe (TAKARA). Expression levels of *eIF4E1* were normalized to the expression level of two reference genes (*ACTIN 2* and *TUBULIN 2*), respectively. The primers used are listed in Supplementary Table S1. Results obtained from three biological repeats with standard deviation (SD).

### Length Analysis of Primary Root, Root Meristem, and Elongation Zone

*eif4e1-1* (CS6552), which was also reported as the *cum1* mutant by Yoshii et al. (2004), and *eif4e1-2* (SALK_145583C) mutants; the complementary line *eif4e1-1*/*eIF4E1*_*pro*_*:YFP-eIF4E1*, which was generated by the cross of *eif4e1-1, eIF4E1*_*pro*_*:YFP-eIF4E1* transgenic line; and the wild-type seeds were surfaced sterilized and germinated on half-strength MS medium, and then grown under normal growth conditions. The length of the primary root of 6-day-old seedlings was measured. The length of RM and elongation zone were measured using 6-day-old seedlings. Data were presented as averages of over 60 seedlings from at least three biological replicates with SD.

### Auxin Treatment

Wild-type (Col-0) seeds were surface-sterilized and germinated on the half-strength MS medium. The total RNA of 7-day-old wild-type seedlings was extracted after 0, 2, 4, and 24 h of treatment with exogenous 10 μM NAA. The expression levels of *eIF4E1* were analyzed by qRT-PCR. In addition, 5-day-old *eIF4E1*_*pro*_*:GUS* transgenic seedlings were transferred to the 1/2 MS liquid medium with or without 10 μM NAA treatment for 12 h, and GUS staining samples were observed under an Olympus BX51 microscope.

### CHX Treatment

Four-day-old seedlings were transferred onto half-strength MS medium containing 50 or 100 nM CHX (Cat: AC466, Genview) for another 3-day growth and then observed or took photos under a confocal microscope.

### Marker Gene Analysis

Reporter lines *DR5*_*rev*_*:GFP, PIN1*_*pro*_*:PIN1-GFP, PIN2*_*pro*_*:PIN2-GFP, PIN3*_*pro*_*:PIN3-GFP, PIN7*_*pro*_*:PIN7-GFP*, and *AUX1*_*pro*_*:AUX1-YFP* were individually crossed with *eif4e1-1*. Homozygous plants were isolated from F2 populations. These homozygotes in F3 and later generations were used for analyses.

### Microscopy

To identify the phenotype of embryos and RM of *eif4e1-1* and *eif4e1-2*, the ovules and seedling roots were cleared in modified Hoyer's solution and an HCG solution as previously described (Chen et al., 2011). The samples were observed by using an Olympus BX51 microscope connected to a Ritiga 2000R camera. Seedlings were observed under a Nikon SMZ1000 microscope equipped with a Nikon digital sight Ds-Fi1 camera.

For confocal microscopy, seedling roots were stained with 100 μg/mL propidium iodide (Sigma-Aldrich) and mounted in 5% glycerol. Images were taken using a Zeiss LSM 780 confocal laser scanning microscope with the following excitation (Ex) and emission (Em) wavelengths (Ex/Em): 488 nm/505–530 nm for GFP, 561 nm/591–635 nm for propidium iodide, and 543 nm/600 nm for FM4-64 as previously described (Huang et al., 2014).

### Yeast Two-Hybrid Library Screening

Screening for interacting proteins of RopGEF7 was performed using a MatchmakerTM Library Construction & Screen Kit (Cat: 630445, Clontech). The cDNA library was generated by using 7-day-old *Arabidopsis* seedlings. The full-length coding sequence of *RopGEF7* was constructed and used as a bait to screen the cDNA seedling library. The cDNA fragments coding for the RopGEF7 were amplified with primer pairs for RopGEF7-F and RopGEF7-R and then cloned into pGBKT7 (BD) to generate the bait of pGBKT7-GEF7. The primers used to generate the constructs are listed in Supplementary Table S1. Seedling cDNA was cloned with pGADT7 (AD) to generate the library. Next, the plasmids of pGBKT7-GEF7 and library were co-transformed into yeast (AH109) following the modified lithium acetate method as directed by the manufacturer. More than 2,000 transformants were screened, and the positive colonies were re-screened on the SD medium lacking Leu, Trp, Ade, and His. The selected clones were further tested on the same medium supplemented with 0 or 10 μM 3-amino-1,2,4-triazole. DNA was extracted from the positive clones and transformed into the *Escherichia coli* strain DH5α to amplify the cDNA inserts, and the plasmids were sequenced.

### Direct Interaction Assays in Yeast Cells

The full-length cDNA, the N-terminal deleted fragment (GEF7-ΔN, aa 60-546), the PRONE domain (GEF7-PRONE, aa 60-447), and the C-terminal fragment (GEF7-C, aa 448-546) of *RopGEF7* were cloned into vector pGBKT7, respectively. The cDNA of *eIF4E1* was cloned into vector pGADT7, respectively. The primers used to generate the Y2H constructs are listed in Supplementary Table S1. The bait and prey constructs were co-transformed by pairs into the yeast AH109, and the transformants were selected on synthetic dextrose (SD) double dropout medium lacking tryptophan and leucine (SD/-Trp-Leu) following incubation at 28°C for 3 days. Single co-transformed yeast clones of double dropout plates were transferred to quadruple dropout medium (SD/-Trp-Leu-Ade-His). The positive (pGBKT7-53/pGADT7-T) and negative (pGBKT7-Lam/pGADT7-T) controls were used.

### Pull-Down Assay

To generate the chimeric eIF4E1 protein, we cloned the full-length cDNA of eIF4E1 to the pGEX-4T-1 vector, which contains a GST tag before the multiple cloning sites (MCS). Next, the fused GST-eIF4E1 protein was expressed in *E. coli* and purified on glutathione affinity resin. The purified fusion protein GST-eIF4E1 was mixed with YFP-tagged RopGEF7 protein (extracted from *35Spro:YFP-RopGEF7* transgenic plants) and then incubated at 4°C for 2 h. The amino acid sequences of GFP and YFP differ only one amino acid, and GFP antibody (Abcam) was used as a substitute for the YFP antibody to detect the YFP-RopGEF7 plant protein. The primers used for plasmid construction are listed in Supplementary Table S1.

### Western Blotting Assay

Plant total protein was extracted as previously described (Chen et al., 2011). Protein samples were separated using 12% sodium dodecyl sulfate (SDS)–polyacrylamide gel electrophoresis and transferred to a polyvinylidene difluoride (PVDF) membrane (Millipore, Billerica, MA, USA). The membrane was incubated with GFP antibody (1:4,000 dilution, Abcam), eIF4E1 antibody (1:330 dilution, customized product at GenScript, USA), or RopGEF7 antibody (1:660 dilution, customized product at GenScript, USA) in TBS buffer (150 mM NaCl, 20 mM Tris–HCL PH 8.0, 0.05% Tween 20) with 5% non-fat dry milk, followed by rabbit horseradish peroxidase (HRP)-conjugated IgG secondary antibody (1:5,000 dilution, Boster Biological Technology Co. Ltd, USA). The signals were exposed to X-ray films using the chemiluminescence detection kit (Thermo-Scientific, USA). Coomassie brilliant blue-stained protein samples were used as loading controls.

### Subcellular Localization and Colocalization of eIF4E1 and RopGEF7

For subcellular localization, the *eIF4E1* coding sequence was inserted into a modified pCAMBIA-1300 vector (*35S:eGFP*) at the *Xba*I site by using a homologous recombination kit (Multis One Step Cloning Kit, VazymE). The *RopGEF7* coding sequence was inserted into the pSAT6-mCherry-C1-B (pE3275) vector containing *35S:mCherry* at the *Sal*I and *Bam*HI sites. Then the resulting constructs were transformed into *Arabidopsis* protoplasts, respectively. GFP and RFP were visualized by using a confocal microscope.

For subcellular colocalization, the aforementioned constructs of *35S:eIF4E1-GFP* and *35S:mCherry-RopGEF7* were co-transformed into *Arabidopsis* protoplasts and then observed by using a confocal microscope.

### Bimolecular Fluorescence Complement Analysis in *Arabidopsis* Protoplasts

To construct the fusion proteins of nYFP-eIF4E1 and cYFP-RopGEF7, the eIF4E1 coding sequence was inserted into the pSAT6-nEYFP-C1 vector, and the RopGEF7 coding sequence was inserted into the pSAT6-cEYFP-C1 vector (Chen et al., 2011). The resulting constructs were co-transformed into *Arabidopsis* protoplasts for BiFC assays. Protoplast isolation and transfection were performed as described (Tao et al., 2005; Chen et al., 2011). YFP was visualized by using an Olympus BX51 microscope using YFP filter sets as previously described (Chen et al., 2011; Huang et al., 2018). The FM4-64 dye labels the plasma membrane. The primers used for plasmid construction are listed in Supplementary Table S1.

### Statistics

All the data were represented the average with SD from at least three biological replicates. A significant difference was determined by paired two-tailed Student's *t*-tests. *P* < 0.05 was considered significant.

### Accession Numbers

The data that support the findings of this study are available in the Supplementary Data section of the online version of this article. Sequence data used in this work can be found in the *Arabidopsis* Genome initiative or GenBank/EMBL databases under the following accession numbers: *eIF4E1* (At4g18040), *eIF(iso)4E* (At5g35620), *RopGEF1* (At4g38430), *RopGEF4* (At2g45890), *RopGEF6* (At3g55660), *RopGEF7* (At5g02010), *PIN1* (At1g73590), *PIN2* (At5g57090), *PIN3* (At1g70940), *PIN7* (At1g23080), *AUX1* (At2g38120), *ACTIN 2* (At3g18780), and *TUBULIN 2* (*AT5G62690*).

## Data Availability Statement

The datasets presented in this study can be found in online repositories. The names of the repository/repositories and accession number(s) can be found in the article/Supplementary Material.

## Author Contributions

TL, QL, and L-ZT: designed the research. TL, QL, HL, JY, and L-ZT: analyzed the data. TL, QL, ZY, CW, HM, GL, RL, GP, and DC: performed the experiments. TL: wrote the manuscript. L-ZT: revised the manuscript. All authors contributed to the article and approved the submitted version.

## Funding

This work was supported by grants from the National Natural Science Foundation of China (32070320, and 31600217), the Natural Science Foundation of Guangdong Province (2019A1515011556 and 2018A030313931), and the Scientific Research Foundation of the State Key Laboratory for Conservation and Utilization of Subtropical Agro-bioresources (SKLCUSA-a201916).

## Conflict of Interest

The authors declare that the research was conducted in the absence of any commercial or financial relationships that could be construed as a potential conflict of interest.

## Publisher's Note

All claims expressed in this article are solely those of the authors and do not necessarily represent those of their affiliated organizations, or those of the publisher, the editors and the reviewers. Any product that may be evaluated in this article, or claim that may be made by its manufacturer, is not guaranteed or endorsed by the publisher.
