# Supplementary material for: eIF4E1 Regulates Arabidopsis Embryo Development and Root Growth by Interacting With RopGEF7
Source: Front Plant Sci. 2022 Jun 30;13:938476. doi: 10.3389/fpls.2022.938476 (PMC9280432; doi:10.3389/fpls.2022.938476)
Supplement: Supplementary file 1 [file Data_Sheet_1.pdf]

SUPPORTING INFORMATION

eIF4E1 regulates *Arabidopsis* embryo development and root growth by interacting with RopGEF7

Taibo Liu, Qianyu Liu, Zhen Yu, Chunling Wang, Huafu Mai, Guolan Liu, Ruijing Li, Gang Pang, Dingwu Chen, Huili Liu, Jiangyi Yang, Li-Zhen Tao

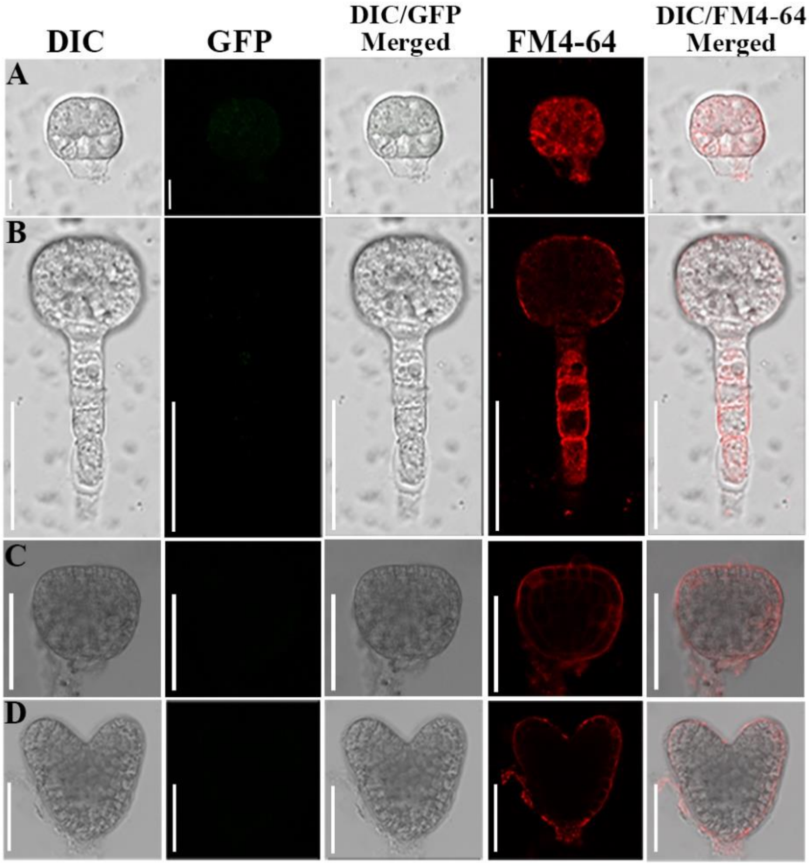

Figure S1. No GFP background signal in wild type embryos

No GFP fluorescence can be seen in 16-cell (A), globular (B), triangle (C), and heart (D) embryo stages of the wild type. The outlines of the embryos were stained by FM4-64 in red in image A to G, respectively. Scale bars: (A) 10 μm; (B-D) 50 μm.

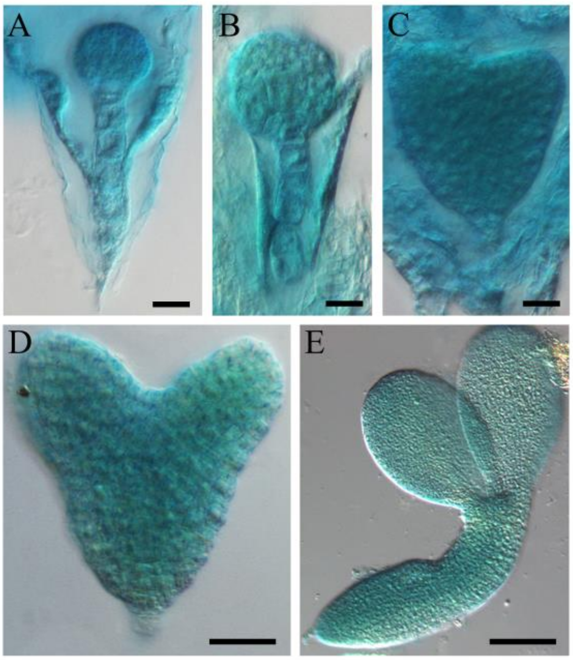

Figure S2. Expression profiles of *eIF4E1$_{pro}$:GUS* in *Arabidopsis* embryos

(A) Early globular, (B) globular, (C) early heart, (D) heart and (E) mature embryo stages. Scale bars: (A-C) 20 μm; (D, E) 50 μm.

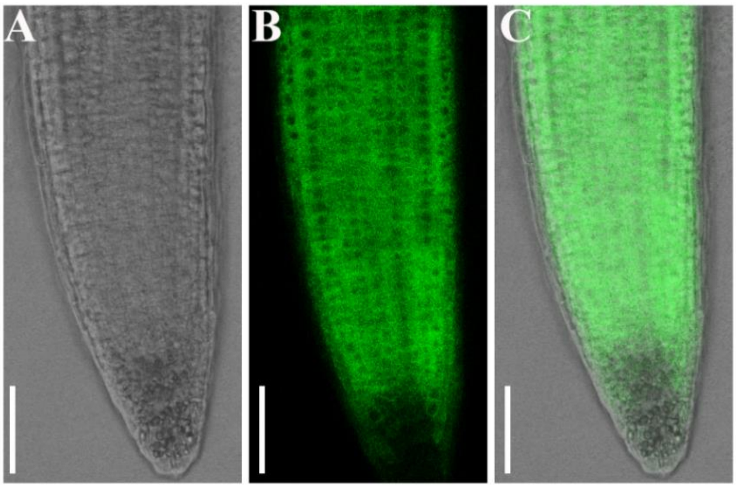

Figure S3. Subcellular localization of YFP-eIF4E1 fusion protein in the roots of four-day-old *eIF4E1$_{pro}$:YFP-eIF4E1* transgenic plants

YFP-eIF4E1 is predominantly localized at cytoplasm. (A) Bright field; (B) GFP; (C) Merged. Scale bars: 20 μm.

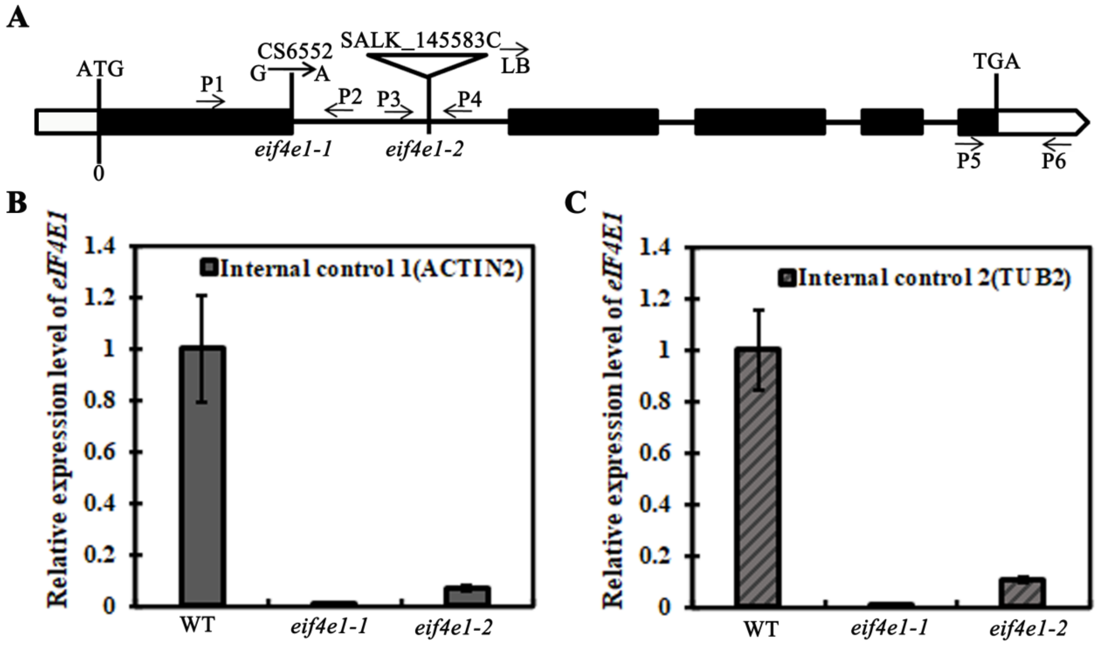

Figure S4. Knock-out or knock-down mutants of *eIF4E1*

(A) Structure of *eIF4E1*. Black and white boxes indicate exons and UTRs, respectively. The introns were displayed as black thin lines between exons. Two mutant lines, the one (*eif4e1-1*, CS6552) is single base replacement mutant (G to A) at 297 bp from the start code in the first exon resulting in tryptophan (Trp, TGG) to stop code (TGA), the other one (*eif4e1-2*, SALK_145583C) harboring a T-DNA insertion in the first intron. The primer pairs P1/P2, and P3/P4 were designed for identifying the mutations. (B-C) Relative mRNA expression levels of *eif4e1-1* and *eif4e1-2* compared to wild type. The primer pair P5/P6 was designed for real-time PCR. Transcription levels were normalized to *ACTIN 2* (Fig. S1B) or *TUBULIN 2* (Fig. S1C), respectively. Data were presented as mean values of three biological repeats with SD.

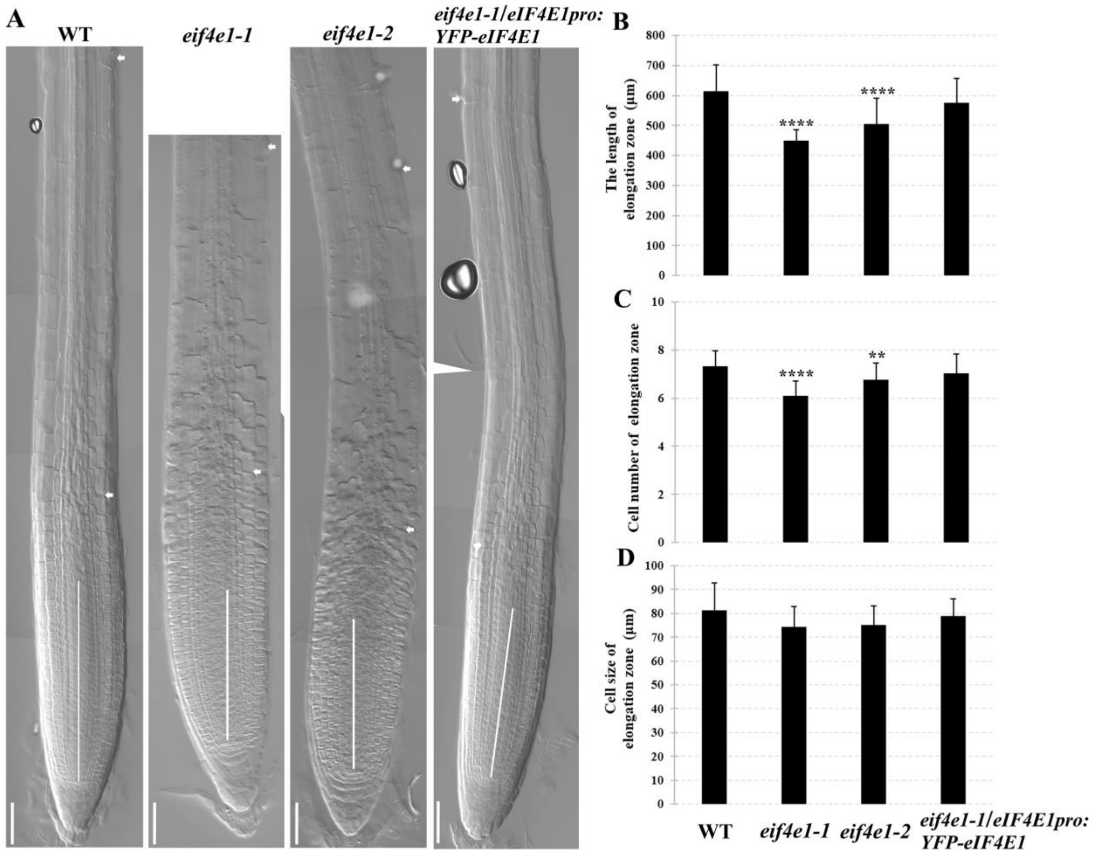

Figure S5. *eIF4E1* mutations affect the length of elongation zone

(A) Comparison of RM and elongation region of six-day-old seedlings among wild type, *eif4e1-1*, *eif4e1-2*, and *eif4e1-1/eIF4E1*$_{pro}$*:YFP-eIF4E1*. In order to show the meristem zone and entire elongation zone, Figure S5A is generated by three to four field photographs (using the Photoshop6 software overlap function). White lines in the middle of root tips indicate the length of RM. The region between two white arrowheads indicates the elongation zone. (B) The length of elongation zone. (C) Cell number of elongation zone. (D) Cell size of elongation zone. Data were presented as mean values with SD, n>30. The asterisks indicate significant difference by Student's *t* test (*P<0.05, **P<0.01, ****P<0.0001). Scale bars: 50 μm.

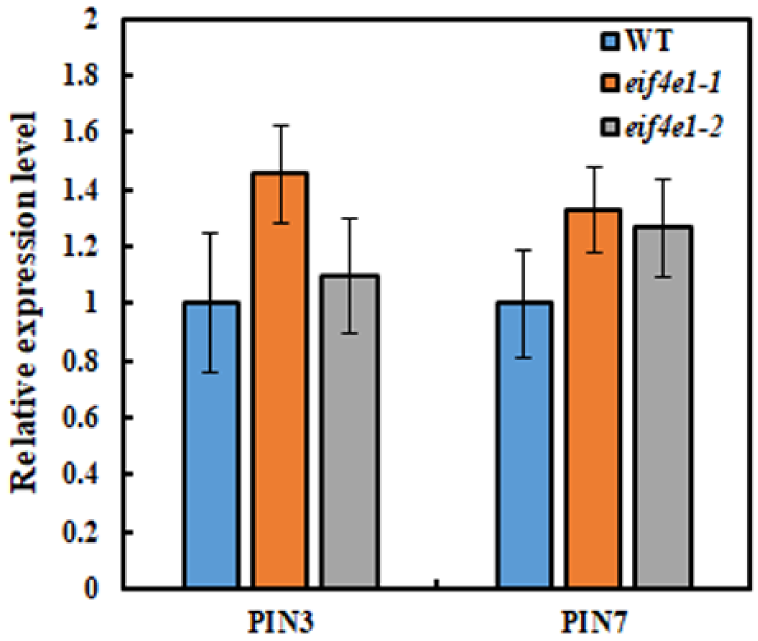

Figure S6. The relative expression levels of *PIN3* and *PIN7* in *eif4e1* mutants and wild type

Transcription levels were normalized to *ACTIN 2*. Data were presented as mean values of three biological repeats with SD.

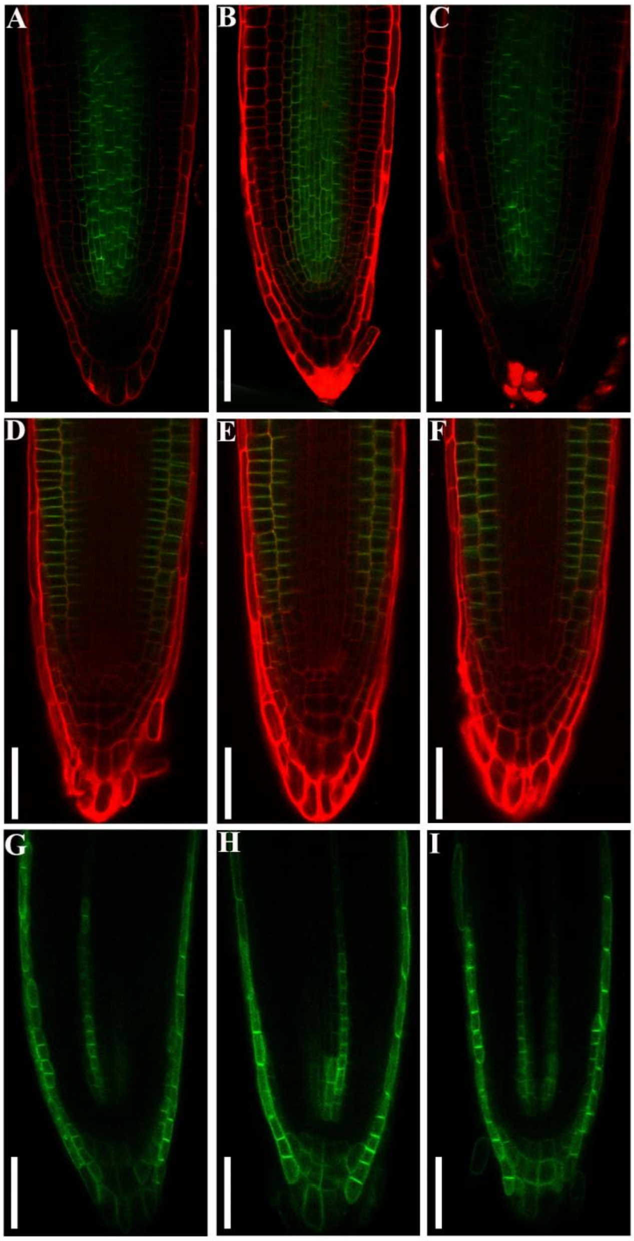

Figure S7. *eIF4E1* mutation does not affect the accumulation of PIN1-GFP, PIN2-GFP and AUX1-YFP

(A-C) $PIN1_{pro}$*:PIN1-GFP* is expressed in roots of four-day-old wild type (A), *eif4e1-1* L1 (B) and *eif4e1-1* L2 (C); (D-F) $PIN2_{pro}$*:PIN2-GFP* is expressed in roots of four-day-old wild type (D), *eif4e1-1* L1 (E) and *eif4e1-1* L2 (F); and (G-I) $AUX1_{pro}$*:AUX1-YFP* is expressed in roots of four-day-old wild type (G), *eif4e1-1* L1 (H) and *eif4e1-1* L2 (I). L1 and L2 indicate two individual lines. Scale bars: 20 μm.

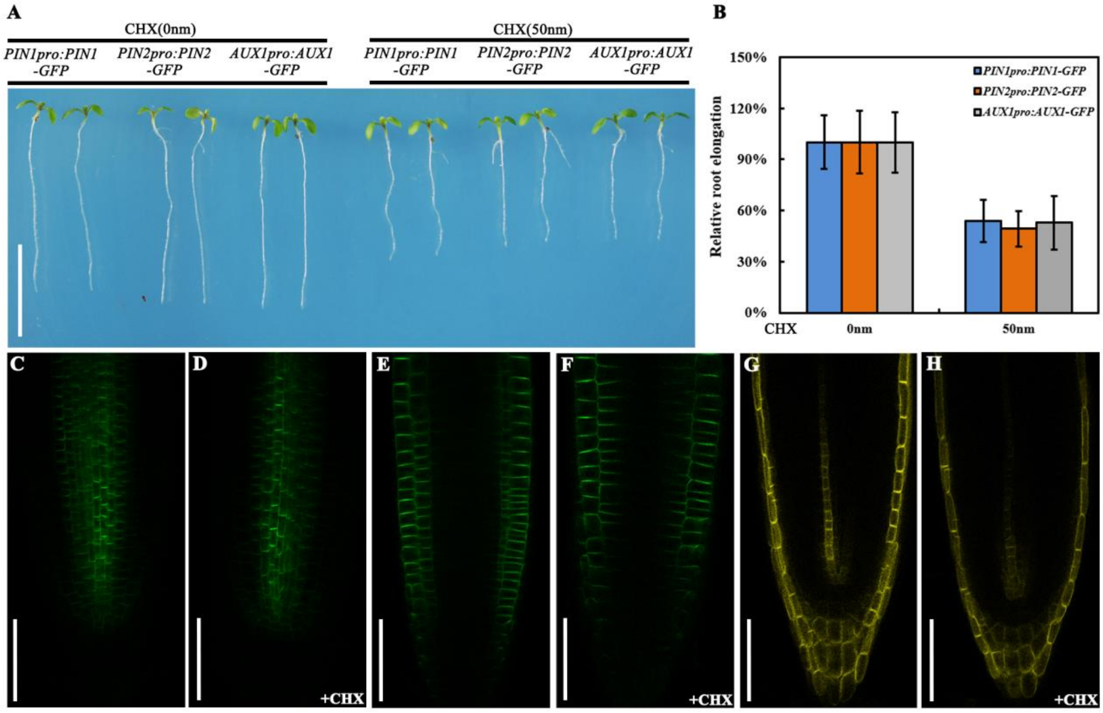

Figure S8. CHX treatment does not affect the abundance of PIN1-GFP, PIN2 -GFPand AUX1-YFP fusion proteins

(A) Four-day-old seedlings of *PIN1*$_{pro}$*:PIN1-GFP*, *PIN2*$_{pro}$*:PIN2-GFP* and *AUX1*$_{pro}$*:AUX1-YFP* were transferred onto half-strength MS medium with or without 50 nM CHX for another three-day growth. (B) The relative root elongation of seedlings in the experiments of Figure S8A. Data were presented as mean values of three biological repeats with SD. (C-H) CHX treatment does not affect the abundance of PIN1, PIN2 and AUX1 proteins in roots of seedlings in the experiments of Figure S8A. (C, D) *PIN1*$_{pro}$*:PIN1-GFP* were treated with (D) or without (C) CHX. (E, F) *PIN2*$_{pro}$*:PIN2-GFP* were treated with (F) or without (E) CHX. (G-H) *AUX1*$_{pro}$*:AUX1-YFP* were treated with (H) or without (G) CHX. Scale bars: (A) 1 cm; (C-H) 50 µm.

Table S1. List of primers used in this study

Experiment	Primer name	Sequence
Vector construction	eIF4E1p-GUS-F	AAGCTTTCTTTCTTTTCTTTCCTCCTCTTTT
	eIF4E1p-GUS-R	GGATCCTGTTTCTCCGAACTGCTTCTCTT
	YFP-F	AAGCTTACAACTGCAGATGGTGAGCAAGGG
	YFP-R	CCCGGGAAAAGGATCCTCTCTTGTACAGCTCGTC
	eIF4E1$_{Pro}$-F	AAGCTTAGGGAAGGTTATTGCGTAGCAAGA
	eIF4E1$_{Pro}$-R	CTGCAGTGTTTCTCCGAACTGCTTCTCTTTC
	eIF4E1 CDS-F	GTCGACATGGCGGTAGAAGACACTCCCA
	eIF4E1 CDS-R	CCCGGGTCAAGCGGTGTAAGCGTTCTTTG
	eIF(iso)4E CDS-F	CATATGATGGCGACCGATGATGTGAAC
	eIF(iso)4E CDS-R	GAATTCTCAGACAGTGAACCGGCTTCTT
	RopGEF7-F	GAATTCATGGATGGTTCGTCGGAAAA
	RopGEF7-R	GTCGACTCAAATCCCAGGATCAAGGTTC
	GEF7-PRO-F	GAATTCAAGAAGATTCGTTCGGATTCA
	GEF7-PRO-R	GTATACTCAGTTGTTGCCTAATGTTGTAGGAA
	GEF7-△N-F	GAATTCAAGAAGATTCGTTCGGATTCAAGA
	GEF7-△N-R	GTCGACTCAAATCCCAGGATCAAGGTTC
	GEF7-C-F	ATCTCAGAGGAGGACCTGCATATGGGGAATGACGCTCCTAAGAG
	GEF7-C-R	GTCGACGGATCCCCGGGAATTCTCAAATCCCAGGATCAAGGTTC
	GEF1-CDS-F	GAATTCATGGGGAGCTTATCTTCTGAGGA
	GEF1-CDS-R	GTCGACATCTCTTTCCGGCGTCACTCCC
	GEF1-PRO-F	GGAATTCCATATGGCAGATGTGGAGATGATGAAGGAGA
	GEF1-PRO-R	CGGGATTCGGTTGCTTTGTTAAGTCGTCCACGTAG
	GEF4-CDS-F	CTGATCTCAGAGGAGGACCTGCATATGATGGAGAGTTCTTCGAATTCCGACC
	GEF4-CDS-R	CAGGTCGACGGATCCCCGGGAATTCCTAATCATCTCTGTTTCTCACTGTTCTG
	GEF4-PRO-F	CTGATCTCAGAGGAGGACCTGCATATGGCAGAGCTAGAGATGATGAGGGAAA
	GEF4-PRO-R	CAGGTCGACGGATCCCCGGGAATTCGTTTCTCACTGTTCTGTCGACGT
	GEF6-CDS-F	GAATTCATGGAGGATAATAGCTGTATCGGGT
	GEF6-CDS-R	GTCGACACCCCGGAGATAATTGGCCAATGCT
	GEF6-PRO-F	ATGGCCATGGAGGCCGAATTCTCAGAGATTGAGTTGTTGAAAGAGA
	GEF6-PRO-R	TCGACGGATCCCCGGGAATTCATCTTTGCTGATG

		TCATCCATGAAC
	35S-mCherry-GEF7-F	AGCTTCGAATTCTGCAGTCGACATGGATGGTTCGTCGGAAAATT
	35S-mCherry-GEF7-R	CGGACTCTAGATCAGGTGGATCCTCAAATCCCAGGATCAAGGTTCG
	35S-eIF4E1-eGFP-F	AGAACACGGGGGACTCTAGAATGGCGGTAGAAGACACTCC
	35S-eIF4E1-eGFP-R	GCCCTTGCTCACCATTCTAGAAGCGGTGTAAGCGTTCTTTG
T-DNA mutant identification	*eif4e1-1*-F	CGTTTTCAACTGTTGAGGAATTCTG
	eif4e1-1-R	TCTAATCCCCCAATAAAGAACATAA
	eif4e1-2-F	TTCCATTGTTTTCCAATGCTC
	eif4e1-2-R	GAAACAAACCTCTTGGGGAAG
	LBb1.3	ATTTTGCCGATTTCGGAAC
Real-time PCR	*eIF4E1* qPCR-F (P5)	GAACGCTTACACCGCTTGAAA
	eIF4E1 qPCR-R (P6)	TTCGTGAACAATCAACACTAGCAA
	qPIN3_F	AAGGAATTGTGCCCTTTGTG
	qPIN3_R	TCGGAAGCGCTATAAGCATT
	qPIN7_F	CGGGGAAGAAGAGTCGGAGAGG
	qPIN7_R	GCAACAAGAGCCCAAATGAGACCA
	ACTIN 2-F	ATGGCTGAGGCTGATGATATTCAAC
	ACTIN 2-R	TACAAGGAGAGAACAGCTTGGATG
	TUBULIN 2-F	ACAAACACAGAGAGGAGTGAGCA
	TUBULIN 2-R	ACGCATCTTCGGTTGGATGAGTGA

Table S2. Quantitative analysis of embryonic phenotype of *eif4e1-1* and *eif4e1-2*

**Genotype**	**8-cell**	**globular**	**transition**	**heart**	**Defect/ Total**	**Percentage（%）**
WT	1/31	0/66	0/12	1/58	2/167	1.20%
eif4e1-1	6/70	15/99	1/21	3/62	25/252	9.92%
eif4e1-2	5/67	13/118	1/23	4/51	23/259	8.88%

Table S3. Seedling defect rate analysis of *eif4e1-1* and *eif4e1-2*

**Genotype**	**Cotyledon defect number / total**	**Defect rate（%）**
WT	1/162	0.62
eif4e1-1	13/168	7.74
eif4e1-2	10/146	6.8
